# Molecular states and spin crossover of hemin studied by DNA origami enabled single-molecule surface-enhanced Raman scattering†

**DOI:** 10.1039/d2nr03664a

**Published:** 2022-10-28

**Authors:** Anushree Dutta, Kosti Tapio, Antonio Suma, Amr Mostafa, Yuya Kanehira, Vincenzo Carnevale, Giovanni Bussi, Ilko Bald

**Affiliations:** Institute of Chemistry, Physical Chemistry, University of Potsdam Karl-Liebknecht-Str. 24-25 14476 Potsdam Germany ilko.bald@uni-potsdam.de anushreedutta@uni-potsdam.de; Dipartimento di Fisica, Università degli Studi di Bari, and INFN, Sezione di Bari via Amendola 173 70126 Bari Italy; Institute for Computational Molecular Science, Temple University Philadelphia PA 19122 USA; Scuola Internazionale Superiore di Studi Avanzati Via Bonomea 265 Trieste 34136 Italy

## Abstract

The study of biologically relevant molecules and their interaction with external stimuli on a single molecular scale is of high importance due to the availability of distributed rather than averaged information. Surface enhanced Raman scattering (SERS) provides direct chemical information, but is rather challenging on the single molecule (SM) level, where it is often assumed to require a direct contact of analyte molecules with the metal surface. Here, we detect and investigate the molecular states of single hemin by SM-SERS. A DNA aptamer based G-quadruplex mediated recognition of hemin directs its placement in the SERS hot-spot of a DNA Origami Nanofork Antenna (DONA). The configuration of the DONA structure allows the molecule to be trapped at the plasmonic hot-spot preferentially in no-contact configuration with the metal surface. Owing to high field enhancement at the plasmonic hot spot, the detection of a single folded G-quadruplex becomes possible. For the first time, we present a systematic study by SM-SERS where most hemin molecule adopt a high spin and oxidation state (III) that showed state crossover to low spin upon strong-field-ligand binding. The present study therefore, provides a platform for studying biologically relevant molecules and their properties at SM sensitivity along with demonstrating a conceptual advancement towards successful monitoring of single molecular chemical interaction using DNA aptamers.

## Abstract 2

The study of biologically relevant molecules and their interaction with external stimuli on a single molecular scale is of high importance due to the availability of distributed rather than averaged information.

## Graphical abstract

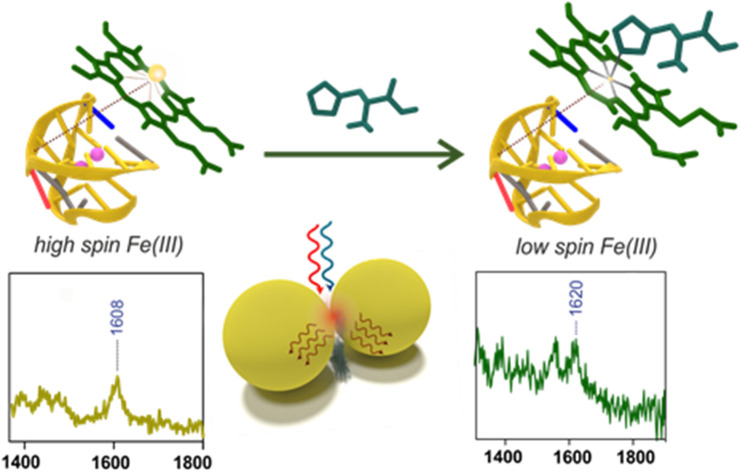

## 1. Introduction

Surface enhanced Raman scattering (SERS) has made considerable advancement in the detection of biologically relevant biomolecules and in understanding their properties, which plays an important role in physiological processes.^1–4^ This is because SERS allows one to obtain direct chemical and structural information in terms of the vibrational fingerprint without the need for molecular labelling.^5^ In that context, single molecule (SM) SERS offers to extract valuable information unique to individual molecules providing leverage over the averaged information obtained from ensemble study.^6,7^ Therefore, the study of reactions or interactions on a SM level can unravel underlying mechanisms prevalent in several redox and catalytic processes.^8–10^ A prerequisite for such SERS based technique is the precise positioning of biomolecules at the plasmonic hot-spot volume which offers highest field enhancement to attain maximum Raman scattering signal required for detection with single molecule sensitivity.^5,11,12^ Among the plethora of SERS-active nanostructures available, DNA origami based plasmonic nanostructures have been unique in demonstrating their utility for detection of dyes, proteins and other important molecules on a single molecule level.^12–21^ This arises due to the unique addressability offered by the DNA staples in the DNA origami design that offers nanoscale precision to position biomolecules of interest at the hot-spot of assembled plasmonic nanostructures.^13,22–25^ Parallelly, smart hot spots generated *via* other chemical and technical means have also shown strong potential to detect important biological molecules like bovine serum albumin (BSA), haemoglobin, polypeptides, and single DNA bases with single molecule sensitivity.^2,26–31^

While single molecule SERS of heme proteins has been demonstrated,^13^ a detailed investigation of the iron-protoporphyrin IX unit – “*hemin*” in terms of molecular state and its molecular interaction to external stimuli in single molecule scale is missing. Hemin – a protoporphyrin IX unit which contains a central ferric iron (Fe^3+^) ion with a coordinating chloride ligand exist in high spin state and forms the basis of many fundamental biological processes.^32^ Additionally, an axial coordination between the active site of metalloenzymes and biomolecules like amino acids in natural enzymes plays an important role in regulating biological phenomena.^8,33,34^ Such interaction is often accompanied by change in oxidation or spin state which induces catalytic cascade reactions.^35,36^ Therefore, understanding and characterization of such complex system and their molecular interaction to external stimuli in sub-nanomolar to single molecule level is important. Therefore, we choose hemin as a model molecule and carry out its study and characterization based on the G-quadruplex–hemin complexation chemistry. The appearance of marker vibrational Raman bands characteristic of hemin oxidation and spin states along with other representative vibrational bands – allows detailed investigation of the system by the SERS technique.^4,36,37^

**Fig. 1:**
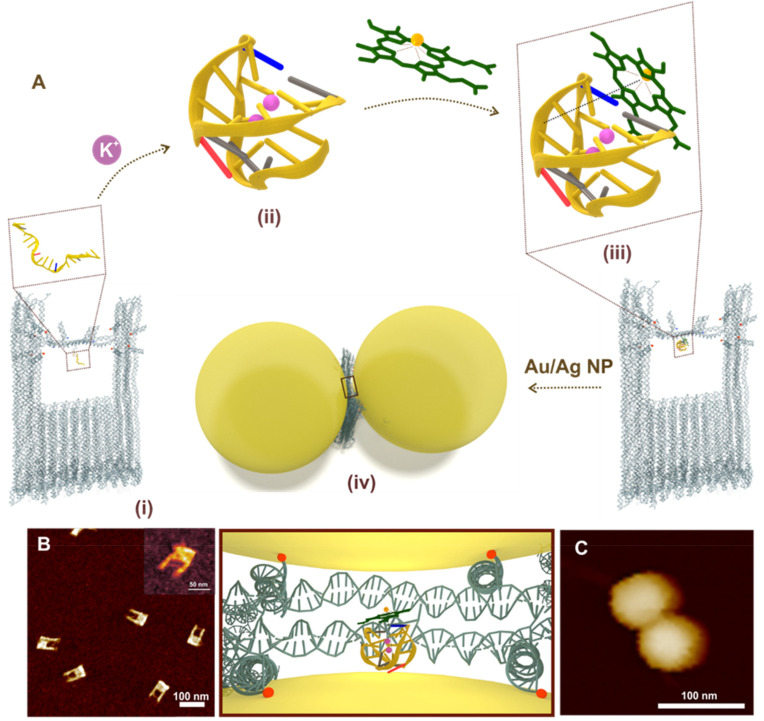
(A) Schematic representation of G-quadruplex (*via* aptamer folding) directed hemin recognition and fabrication of Au/Ag DONAs – (i) DNA origami nanofork with aptamer in the bridge (zoom-in view of aptamer in box); (ii) folded G-quadruplex structure in presence of K^+^ ions in the bridge where nucleobase(s) in blue, grey, red and yellow represent cytosine, thymine, adenine and guanine, respectively. The backbone is represented as the yellow line connecting the bases; (iii) complexation of hemin (represented in green) with the folded G-quadruplex in the bridge – the dashed brown line represents the electrostatic interaction between Fe^3+^ centre with negative charge of oxygen in the phosphate of sugar-phosphate backbone. (iv) Nanoparticle (Au/Ag) coated with differently functionalised DNA sequence hybridized with DNA capture strand (end labelled with red spheres for representation) hanging out from the upper arms and bridge position (blue spheres) of the DNA origami nanofork to form DONAs. Brown box shows the zoom in top-view of the G-quadruplex–hemin complex bound at the plasmonic hot-spot of DONAs. (B) AFM image of the folded nanoforks with PS2.M aptamer in the bridge, with DNA bridge clearly visible (inset shows the close up view of a DNA origami nanofork with DNA bridge). (C) Representative AFM image of a Au DONA.

It has been well argued and established in literature that successful SM measurements are guided by the confined picocavities that is formed during near field measurements, which involves direct metal–molecule interaction.^38^ In that regard, equally important is to utilize a potential SERS substrate that makes the study of such complex system feasible in their native state by limiting the contact with the molecule of interest. For this, we employ a recently reported DNA origami nanofork antenna (DONA) (Fig. 1A(i); Fig. S1, ESI†) that offers high SERS signal enhancement up to 10^9^–10^11^ and has shown promising results for single molecule detection.^13^ A uniquely addressable DNA staple strand located in the centre of the DNA nanofork bridge allows the positioning of a DNA aptamer specific to hemin trapped at the hot-spot.

Therefore, within this report, we investigated the following fundamental questions: (i) can we characterize single G-quadruplexes, their structure and its complex with hemin by SERS to gain information on its molecular state using DONAs? (ii) Can SERS results help us to comment on the orientation of a single G-quadruplex in the plasmonic hot spot? (iii) Does the molecule need to be in contact with the plasmonic surface to obtain signal at SM scale? What is the utility of the DONA structures in this aspect? (iv) Which plasmonic nanostructures (gold (Au)/silver (Ag)) serve as suitable SERS probe for the hemin recognition and molecular interaction studies? (v) Further, can we record the spin state crossover of hemin upon strong field ligand addition as an example of a single molecule chemical interaction study?

## 2. Experimental section

### 2.1. DNA origami nanofork folding with PS2.M hemin aptamer modification

The nanofork DNA origami structure used for the experiment is the same as reported in our previous report.^13^ ssDNA staples and scaffold strands were purchased from Metabion and Integrated DNA Technologies (IDT) respectively. All staple sequences are the same as reported in ref. 13 for the nanofork DNA origami, except for the modifications mentioned in Table S1, ESI† for the designated position. The self-assembly process was carried out by mixing 67.5 μL Milli-Q water, 10 μL buffer (10× TAE, 150 mM MgCl_2_), 19 μL staple solution (individual concentration of 200 staples in the solution = 0.5 μM), 2.5 μL scaffold (m13mp18, 100 nm) and 1 μL modified PS2.M aptamer (100 μM) staple in a PCR tube. This was then subjected to annealing process in a thermocycler for 12 h. The folding solution was first heated to an initial temperature of 80 °C and gradually cooled down in steps of 12 min to 20 °C for over 12 h.

The folded DNA origami nanofork structures were purified against unreacted ssDNA sequences using 100 kDa centrifugation filters (Amicon, Merck, Centrifugal filter, 100 kDa) in a series of three washing cycles at a speed of 7000 rpm for 5 min (20 °C) each. The remaining volume in the 100 kDa filter was collected and the concentration of the TAE and MgCl_2_ in the solution was adjusted to 1× and 15 mM respectively. The concentration of the DNA corresponding to the concentration of the DNA origami was calculated using an UV-vis spectrophotometer (Nanodrop). Structural characterization of the folded nanoforks were carried out by atomic force microscopy (AFM) using a Bruker Multimode 8 (Billerica, Massachusetts, US) (Fig. S1 and S2, ESI†). For all sample depositions for AFM, we have used 6 mm × 6 mm silicon chips, which were pre-cleaned in acetone solution followed by thorough wash in water : ethanol (1 : 1) mixture. The cleaned silicon chip was then plasma treated for 7 min prior to sample deposition. 10 μL (8 nM) of freshly prepared nanofork was deposited on a silicon chip and incubated for 10 min at ambient condition. The magnesium concentration in the sample was adjusted to 45 mM using 10× TAE, 150 mM Mg^2+^ buffer prior to deposition. Finally the chip surface was washed with water : ethanol (1 : 1) mixture, blow dried and imaged.

Details of the PS2.M aptamer sequence and the corresponding bridge staple sequence modified with the same is listed in Table S1, ESI.†

### 2.2. Nanoparticle functionalization with ssDNA

Au NPs (citrate stabilized, NanoXact, 60 ± 6 nm, 2.4 × 10^10^ particles per mL) and Ag NPs (citrate stabilized, NanoXact, 59 ± 6 nm, 1.9 × 10^10^ particles per mL) are purchased from NanoComposix (San Diego, CA, USA). The dithiol phosphoramidite (DTPA)-modified ssDNA staples (tabulated in Table S1, ESI†) are purchased from Metabion. Trisodium citrate dihydrate (TSC), Tris (2-carboxyethyl) phosphine (TCEP), sodium dodecyl sulphate (SDS) and sodium bromide (NaBr) are purchased from Sigma-Aldrich (Sigma-Aldrich Chemie GmbH, Munich, Germany).

The functionalization of Au NPs with ssDNA strands ((TTT)_8_T_4_ or (GTT)_8_T_4_) each were followed using a modified salt aging method.^13,39^ For this, 400 μL of Au NPs (60 nm) are taken and centrifuged at 6000 rpm for 5 min at 20 °C and the residue redispersed in 26.5 μL of Milli-Q water. Simultaneously, disulphide bond activation was carried out by treating 4 μL of DTPA modified ssDNA with 1 μL TCEP (100 mM) for 30 min which cleaves the disulphide bond. In the next step, 5 μL of cleaved DNA solution along with 3.5 μL SDS (0.2%) was added to the 26.5 μL Au NPs dispersion and incubated for 90 min at 40 °C. This was followed by NaBr treatment in a step wise manner such that the final concentration of the NaBr in the dispersion is 300 mM where each addition step involved 10 min incubation time at 35 °C. The steps are as follows: the initial first 4 steps involved the addition of 1.7 μL NaBr (0.4 M), followed by addition of 2.1 μL, 2.3 μL and 3.3 μL (×2) of 1 M NaBr in each successive steps. Next step involved the addition of 3.0 μL (×2) and 3.3 μL (×2) of 15 mM MgCl_2_ in a similar step wise manner such that the final concentration of the dispersion is 4.5–5 mM. Finally the dispersion is subjected to three step washing cycles using 5 mM MgCl_2_ and 0.02% SDS solution at 5000 rpm for 5 min each at 20 °C.

The functionalization of Ag NPs with ssDNA strands ((TTT)_8_T_4_ or (GTT)_8_T_4_) each was followed using a pH 3 switch method.^13,39,40^ First, 400 μL of Ag NPs (59 nm) are taken and centrifuged at 6000 rpm for 5 min at 20 °C and the residue redispersed in 40 μL of Milli-Q water. Simultaneously, disulphide bond activation was carried out by treating 4 μL of DTPA modified ssDNA with 1 μL TCEP (100 mM) for 30 min which cleaves the disulphide bond. In the next step, the 5 μL of the above cleaved ssDNA solution was added along with 4.5 μL of 2% SDS to the Ag NP dispersion and incubated for 30 min at 30 °C. Post incubation, the pH of the Ag NPs dispersion was adjusted to 3 by adding 6.5 μL TSC (0.5 M) buffer followed by incubation for 20 min at 30 °C. Post incubation, the Ag NP dispersion was treated with NaCl solution (1 M) in a stepwise manner in an aliquot of 2 μL each time to reach a final concentration of 0.3 M. The mixture was vortexed after each addition of NaCl solution. Finally, the dispersion was subjected to washing steps (×3) using 0.02% and 5 mM MgCl_2_ as mentioned in case of Au NPs.

### 2.3. Folding of G-quadruplex, complexation of hemin with folded G-quadruplex and fabrication of DNA origami nanofork antenna (DONAs)

The G-quadruplex folding was carried out in presence of K^+^ ions.^41,42^ Briefly, 15 μL (11 nM) folded DNA origami nanofork was treated with 4 μL of a 100 mM KCl solution and incubated for 45 min at 40 °C under constant shaking. This resulted in the PS2.M aptamer unit appended to the bridge staple sequence to fold up into G-quadruplex structure.

This was followed by addition of 2 μL (10 μM) freshly prepared hemin (purchased from Sigma Aldrich) solution to the above nanofork solution which was then incubated for 30 min at 37 °C under constant shaking (250 rpm). The final solution was then subjected to three centrifugal cycles with 5 mM Mg^2+^ solution to get rid of the excess potassium ions and hemin molecules (centrifugation conditions: 7000 rpm/5 min/20 °C for first two cycles and 7000 rpm/5 min/20 °C for the third cycle).

The next step involves the Au DONA fabrication where we employ two differently functionalised AuNPs *i.e.*, two different batches of AuNPs – one coated with (TTT)_8_T_4_ and the other with (GTT)_8_T_4_ sequence. The ratio of nanofork : (TTT)_8_T_4_ : (GTT)_8_T_4_ is 1 : 1.5 : 1.5 such that the final concentration of nanofork in the hybrid mixture is 0.08–0.1 nM. The mixture was hybridized in a thermocycler by heating up to 37 °C and gradually cooling it down to 20 °C in steps of 12 min and 50 seconds over a time range of 3.5 h. The as obtained Au DONAs were then further purified from the mixture by running gel-electrophoresis. The Au DONAs (mixed with 30% glycerol in a ratio of 4 : 1) were run in 1% agarose gel made in 5 mM Mg^2+^ solution for 60 min under an applied voltage of 80 V. After the gel run, the dimer band was cut and the Au DONAs were extracted by squeezing out of the agarose using microscopic glass slides. The layer marked in the box shown in Fig. S3, ESI,† refers to the dimer band. Similar steps were followed for Ag DONAs.

Hemin stock solution: A stock solution of 15.3 mM (10 mg mL^−1^) of hemin was prepared in NaOH solution (1 M). A final stock solution of 10 μM was prepared by diluting 10 μL of 100 μM hemin stock solution in water : ethanol (40 : 50) mixture.

### 2.4.A. Sample preparation for AFM imaging of Au and Ag DONAs

AFM imaging of DONAs was carried out using Bruker Multimode 8 atomic force microscope (Billerica, Massachusetts, US). For AFM imaging of Au and Ag DONAs, 10 μL of gel-purified sample dispersion was drop cast on plasma treated Si-chip and the concentration of magnesium was adjusted to 50 mM. The samples were incubated for 45 min followed by washing off the excess sample with water : ethanol (1 : 1) mixture. The samples were then blow-dried and imaged under specified condition.

### 2.4.B. Sample preparation for TEM imaging of Au DONAs

The TEM samples (Au DONAs) were imaged with JEOL JEM 1011 transmission electron microscope (JEOL, Akishima, Tokyo, Japan) equipped with Olympus MegaView G2 camera and using 80 kV acceleration voltage. The copper grids consists of 1 nm carbon layer on top of 10 nm Formvar film (EFCF400-Cu-50, Science Services GmbH, Unterhachinger Straße 75, Munich, Germany). 3 μL of Au DONA solution was dropcast on the grid and incubated for 2–3 min. The excess solution was then blotted away, followed by addition of 3 μL of staining solution. After 2 min incubation, the excess staining solution was blotted away, and the film was washed twice with 3 μL of Millipore water and the excess liquid was blotted away. The grid was then left to dry in room temperature before imaging.

### 2.5. Sample preparation for SERS mapping and measurement of Au/Ag DONAs, control and reference, respectively

For all sample depositions, we have used 6 mm × 6 mm silicon chip which was cross-scratched from the center for reference.

A Witec Alpha 300 Raman microscope (Witec, Ulm, Germany) was used for all SERS measurements. A spectrograph (Blaze 500, grating 600 gr mm^−1^) equipped with an Andor DV401-BV CCD-camera was used for 488 nm laser excitation. For 633 nm, a spectrograph (Blaze 750, grating 600 gr mm^−1^) equipped with Andor DU401A-BR-DD-352 CCD camera was used. The laser power used for Raman mapping and the corresponding integration time per point is specified in the discussion.

(i) SERS measurement/mapping of Au/Ag DONAs with G-quadruplex–hemin at the plasmonic hot-spot: The sample preparation for SERS mapping of as-fabricated Au/Ag DONAs is the same as described in Experimental section 2.4. The SERS mapping of Au DONAs was carried out with 633 nm laser excitation, 250 μW and 100× objective with an accumulation time of 4 s. Conditions for Ag DONAs remains the same except for the laser power, which was 80 μW. The mapping area was varied for each measurement.

(ii) Control SERS measurements of Au/Ag DONAs with PS2.M aptamer only: The DNA origami nanofork with the PS2.M aptamer in the central bridge position was hybridized with two differently functionalized Au/Ag NPs according to the method described in the above section 2.3. Sample preparation for SERS mapping is same as described in Experimental section 2.4.

(iii) Control measurement with Au DONAs fabricated with hemin without PS2.M aptamer: For this, the DNA origami nanofork was folded without the PS2.M aptamer unit. The as-prepared DNA origami nanofork was treated with hemin which was followed by nanoparticle hybridization to generate Au DONAs as described in Methods section 2.3. Sample preparation method for SERS mapping is the same as described in Experimental section 2.4(ii). The SERS mapping of Au DONAs was carried out with 633 nm laser excitation, 250 μW and 100× objective with an accumulation time of 4 s.

(iv) Reference SERS measurement of hemin on AuNPs: For this, 60 nm AuNPs were centrifuged (twice) at 6000 rcf for 5 min at 20 °C to remove excess citrate. The precipitate so obtained was redispersed in 100 μL Milli-Q water to which freshly prepared hemin solution (15.3 mM) was added such that the final concentration of the dispersion is 153 μM. The sample dispersion was allowed to mix at 37 °C under shaking for 2 h. The final mixture was centrifuged twice to remove excess unreacted hemin molecule and the final precipitate redispersed in 100 μL water. 10 μL of the above dispersion is deposited on plasma treated Si-chip and dried overnight and point SERS spectra were collected on selected areas with 633 nm laser excitation as specified in ESI.†

### 2.6. SM SERS analysis from correlated SEM-Raman map of Au DONAs

The SM SERS spectra corresponding to nanoparticle dimers were extracted with the help of correlated Raman-SEM maps. Cross marks on the Si-chip were used to locate the sample area mapped in Raman. Post Raman measurement, the mapped area is imaged in SEM (Thermo Fisher Phenom ProX Desktop SEM or FEI Quanta250). The SEM image is then overlaid on the Raman map. Thereafter, SERS signals from chosen nanoparticle dimers were analyzed. Not all DONAs showed detectable Raman scattering intensity. Importantly, DONAs closely spaced to trimers, tetramers or aggregates were neglected to avoid ambiguity. An example of Raman and SEM correlation is shown in ESI.† Band assignments for all SERS measurements can be found in Tables S2–S4, ESI.†

SM SERS analysis for Ag DONAs and their corresponding control SERS measurements were performed in a similar fashion as described above.

### 2.7. Time series measurements on single DONAs in dark field mode

The time series SERS data has been collected for single Ag DONAs with hemin complexed to G-quadruplex in the center of the DNA origami nanofork bridge. For this a sample area close to the scratch marker on the Si-chip was imaged by AFM to identify single Ag DONAs along with other trimers, tetramers and other aggregates. The same area was then imaged in dark-field mode, which allowed identifying the position of the same single DONAs, and their aggregates spotted in AFM. This was successfully done by matching the particle deposition pattern observed in AFM with the dark field view where DONAs appeared as bright round dots. Thereafter, individual Ag DONA positions were identified and the sample area was moved to the reference laser spot focus area to measure the time series SERS under specified condition.

For this, we used the same Witec Alpha 300 Raman microscope equipped with the dark-field scattering set-up. A Zeiss Hal-100 Halogen lamp (100 W) was used for the dark-field scattering experiment with a 100× BD objective (NA 0.9) from Zeiss EX EPIplan-NEOFLUAR.

### 2.8. Complexation of DNA origami nanofork bound G-quadruplex–hemin complex with external ligand and fabrication of external ligand bound DONA

The G-quadruplex folding was carried out in presence of K^+^ ions as described in section 2.3. This is followed by addition of 2 μL (10 μM) freshly prepared hemin (purchased from Sigma Aldrich) solution to the above nanofork solution which was then incubated for 30 min at 37 °C under constant shaking (250 rpm). The final solution was then subjected to three centrifugal cycles with 5 mM Mg^2+^ solution to get rid of the excess potassium ions and hemin molecules (centrifugation condition: 7000 rpm/5 min/20 °C). This is followed by addition of different ligand solutions (histidine, cyanide and lysine) such that the final concentration of the ligand in the DNA origami nanofork bound G-quadruplex–hemin complex is 1.5 μM. The resulting mixture was stirred for 30 min at 30 °C. The final solution was centrifuged at 7000 rpm/5 min/20 °C during first cycle and 7000 rpm/10 min/20 °C during the second cycle.

Next step involves the Ag DONA fabrication following the same procedure mentioned in section 2.3 followed by gel purification to obtain free Ag DONAs. The steps for sample preparation for SERS measurement remains the same as described in section 2.5(i).

## 3. Results and discussion

The universal configuration for a SERS substrate is the dimer of plasmonic nanoparticles which are synthesized utilizing our recently reported DNA origami nanofork structure.^13^ The proposed DNA origami nanofork has an inbuilt double stranded DNA bridge with a uniquely addressable DNA staple strand located in the centre of the bridge that allows the positioning of a DNA aptamer specific to hemin. Each arm of the DNA origami nanostructure is equipped with four single stranded capture DNA strands (indicated in red dots, Fig. S1, ESI†) and the bridge with two additional capture sequences (indicated in blue dots; Fig. S1†) – both extending from each side of the arm and bridge to hybridize with the complementary capture sequence from the incoming nanoparticles to form the DONA structures in the subsequent steps. More detailed discussion on the DNA origami nanofork structure can be found in ref. 13.

To further pursue the spectroscopic investigation of the iron(iii) protoporphyrin unit – hemin, we have chosen the PS2.M aptamer unit – an *in vitro* selected synthetic DNA oligomer which is folded in presence of K^+^ ions to form a G-quadruplex structure in the bridge.^42^Fig. 1A shows the overall scheme of G-quadruplex directed hemin capture and fabrication of Au DONA. The AFM image in Fig. 1B shows the successful folding of the DNA nanofork with (i) PS2.M aptamer in the bridge. (ii) Addition of K^+^ ions (100 mM) led to the folding of the randomly configured aptamer sequence in the bridge into a G-quadruplex structure.^4,41,43^ More supportive AFM images are shown in Fig. S2, ESI.† (iii) Subsequent addition of hemin in nanomolar concentration directs its complexation with the folded G-quadruplex *via* electrostatic interaction. (iv) The next step involves the hybridisation of the nanofork with 60 nm Au or Ag nanoparticles to generate the nanofork dimer nanostructure abbreviated as DONAs^13^ such that the plasmonic hotspot traps the G-quadruplex–hemin complex. The as obtained Au DONAs were then further purified from the mixture by running gel-electrophoresis (Fig. S3, ESI†). Details of the overall steps (i–iv) can be found in the Experimental section. Fig. 1C shows a representative AFM image of a single Au DONA for reference (More AFM images of Au DONAs in Fig. S4A–D, ESI†). Further, experimental procedures for Ag DONA fabrication and the corresponding AFM images can be found in Experimental section and Fig. S5A–D, ESI† respectively.

**Fig. 2:**
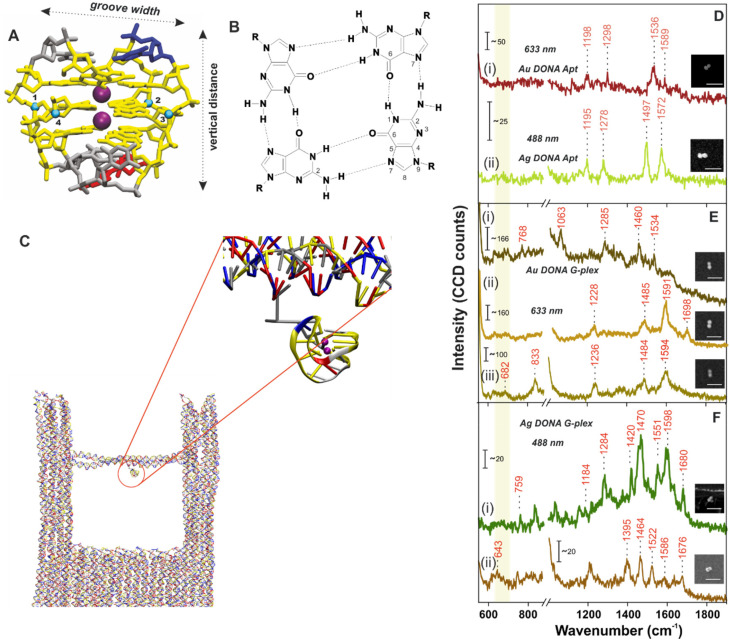
(A) Atomistic model of the G-quadruplex structure. The colour coding matches the one in Fig. 1. (B) Cross sectional view of the guanine tetrad showing the Hoogsteen type H-bond interaction between dG bases. The groove width is computed considering the backbone to backbone distance between the C4′ atoms shown and numbered as blue spheres. (C) Snapshot of the G-quadruplex attached to the nanofork (encircled in red; zoomed in view) from the coarse-grained oxDNA2 molecular dynamics simulation. SM SERS spectra of (D) aptamer bound (i) Ag DONAs and (ii) Au DONAs and that after formation of G-quadruplex using (E) Au DONAs and (F) Ag DONAs. Au DONAs and Ag DONAs in all cases were measured under laser excitation of 633 nm (Obj: 100×; power: 250 μW; acc time: 4 s) and 488 nm (Obj: 100×; power: 80 μW; acc time: 4 s), respectively. Insets in (D–F) show SEM images of the respective DONAs; scale bar = 250 nm and (i), (ii) or (iii) in (E), (F) and (G) represents individual DONA spectra. The yellow vertical line highlights the ring breathing frequency region.

No crystal structure data for PS2.M is available to our knowledge, hence we have first constructed an atomistic model for a specified structure of the G-quadruplex from PS2.M^44,45^ shown in Fig. 2A using the 3D-NuS webserver,^46^ and then performed a 1μs atomistic molecular dynamics simulation of the molecule (refer ESI† for details). Förster resonance energy transfer (FRET) further ensured the successful folding of aptamer upon addition of K^+^ employing coumarin (C343) and fluorescein (6-FAM) as donor–acceptor dye pair^42^ (experimental procedure and results in ESI; Fig. S6†). We first determine the overall dimension (height and G-tetrad groove width; Fig. 2A) of the folded G-quadruplex and its orientation with respect to the nanofork bridge (details in ESI; Fig. S7A–C†). The cross-sectional view of the G-tetrad plane (Fig. 2B) depicts the interbase Hoogsteen hydrogen bonds.^4^ A coarse-grained molecular dynamics simulation carried out using the oxDNA2 model^47,48^ reflected completely random orientation of the G-quadruplex with respect to the nanofork. Snapshot shown in Fig. 2C shows one possible orientation; refer Fig. S8, ESI† for RMSD plot and other details. The average hot-spot gap distance calculated from multiple Au DONAs was 1.7 ± 0.13 nm which is well enough to accommodate the folded G-quadruplex. Exemplary TEM images of Au dimers and their corresponding gap distance is shown in Fig. S9.†

The overall process involving the folding of aptamer to form the G-quadruplex leading to hemin recognition has been tracked by SM SERS measurements using the DONA structures. SM-SERS measurements carried out for Au (633 nm laser) and Ag DONAs (488 nm laser) with aptamer show bands at 1198 cm^−1^, 1298 cm^−1^, 1536 cm^−1^, 1589 cm^−1^ and 1195 cm^−1^, 1278 cm^−1^, 1497 cm^−1^, 1572 cm^−1^, respectively.^4,49^ Details of the band assignment can be found in ESI (Table S2†). The peaks appearing at 1198 cm^−1^ and 1195 cm^−1^ for the Au and Ag DONA (Fig. 2D(i) and (ii)) structures can be attributed to deoxythymidine unit. Simultaneously, the peak at 1278 cm^−1^ represents the ring stretching and C–H ring bending of thymine observed in Ag DONAs which however appears shifted to 1298 cm^−1^ for Au DONAs. The unfolded state of the aptamer is quite clear from the peak at 1497 cm^−1^ (Fig. 2D(ii)) which corresponds to the C8=N7 and of G when it is not H-bonded.^4^ However, the peak at 1572 cm^−1^ corresponds to interbase deformation (*δ*) of dG N2H indicating the partially folded conformation of the aptamer in some cases (Fig. 2D(ii)).

In the next step, K^+^ addition (50 μM) to the nanofork dispersion resulted in the formation of G-quadruplex, which is reflected in the SM-SERS spectra of Au and Ag DONAs respectively (Fig. 2E and F). The frequency lowering of the C8=N7 band of guanine that appears around 1497 cm^−1^ for the native aptamer down to 1485 cm^−1^ (due to C8=N7–H2 deformation) is characteristic of G-quadruplex formation, indicative of strong inter-base Hoogsteen H-bonding interaction (Fig. 2E(ii) and (iii) for example). The diagnostic peak around 1484 ± 1.8 cm^−1^ (appearing in normal Raman spectra) of the guanine tetrad occasionally showed a further red-shift in SM-SERS to the range 1463 ± 2.5–1471 ± 2.1 cm^−1^ which can be seen in Fig. 2E(i) in case of Au DONAs and Fig. 2F(i) and (ii) for Ag DONAs.^49^ This apparent red-shift from normal Raman wavenumber could be attributed to near surface orientation of the dG N7 Hoogsteen hydrogen bond or intermediate surface interaction.^50^ In turn, the appearance of SERS bands at wavenumbers corresponding to normal Raman scattering is indicative of the G-quadruplex in non-contact configuration at the hot-spot. Details of the band assignment can be found in ESI (Table S3†). Based on the MD simulation, the G-quadruplex attached to the bridge can attain random orientation. However, the overall analysis of individual DONAs indicates the preferential orientation of G-tetrad parallel to the nanoparticle surface due to lower probability of the appearance of ring breathing mode in the region 640–660 cm^−1^.^49,51,52^

Additionally, the vibrational band observed in case of Au DONAs at 682 cm^−1^ (Fig. 2E(iii)) is indicative of guanosine in C2′-*endo*/anti conformation expected in anti-parallel G-quadruplex configuration.^4^ This is further supported by the peak at 1395 cm^−1^ in case of Ag DONA (Fig. 2F(ii)) that corresponds to C2′-*endo*/anti deoxy thymidine.^4^ Additionally, new peaks arising at 1698 cm^−1^ (Fig. 2E(ii)), 1680 cm^−1^ and 1676 cm^−1^ (Fig. 2F(i) and (ii)) resulting from strong H-bonding between C6=O6 and H1 was further indicative of the G-quadruplex formation.^51^ Detailed peak assignment of the G-quadruplex can be found in Table S3, ESI.† To our knowledge, for the first time the Raman shifts following single G-quadruplex formation have been characterized along with the spectral band interpretation.

To be noted, (i) SM SERS spectral analysis of different Au/Ag DONA structures revealed peak wandering of the characteristic peaks as mentioned above. (ii) In addition, certain vibrational bands observed for one DONA structure (Au/Ag) are found absent in the spectra of the other DONA structures. (iii) Also, most of the bands could be assigned using normal Raman wavenumber while only few peaks showed up at typical SERS wavenumber position. This can be explained as follows: The as-designed configuration of the DONA allows the target molecule to be bound to the bridge without inducing direct contact with the surrounding nanoparticles. This is indicated from the band at 1485 cm^−1^ (due to C8=N7–H2 deformation) which appears at lower wavenumber when oriented near to the surface. This also makes room for higher degree of fluctuation freedom attained by the G-quadruplex conformation at the hot-spot (or, target molecule in the bridge). Under such condition, the long-range electromagnetic field enhancement (within tens of angstroms at the hot-spot) allows the successful detection of the vibrational fingerprint observed.^53^ Nevertheless, the probability of appearance of vibrational bands and the respective shift observed will rely on their relative near surface orientation and attained conformation which will boost the Raman signal corresponding to any particular functional group and/or intrastrand structure.^5,52,53^ Equally possible could be that the G-quadruplex or the target unit, owing to the fluctuation freedom might come in near contact to the surface which will result in vibrational bands to appear at SERS wavenumber position as already observed above.^50^ The same phenomenon will also be seen in the rest of the analysis which we interpret by the mention of Raman and SERS wavenumber in brackets. The SM SERS spectra shown in Fig. 2D–F and in the rest of the manuscript are obtained by correlated Raman-SEM technique described in Experimental section and as an example shown in Fig. S10, ESI.†

**Fig. 3:**
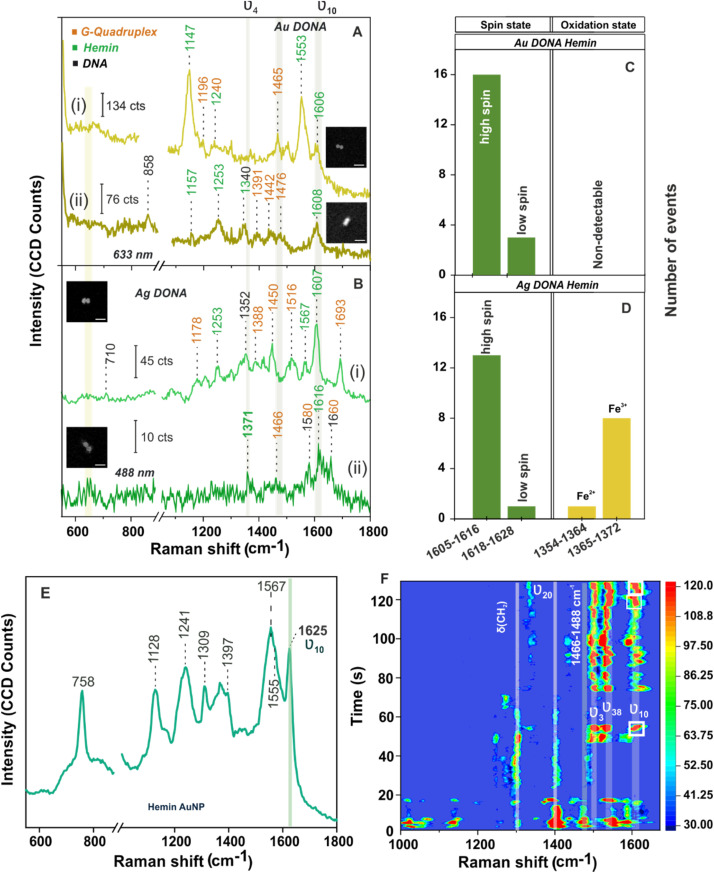
SM-SERS spectra of hemin bound (A) Au DONAs and (B) Ag DONAs under laser excitation of 633 nm and 488 nm, respectively (inset shows SEM image of the corresponding DONAs; scale bar = 125 nm; measurement condition – obj: 100×; power: 250 μW (Au) and 80 μW (Ag); acc time: 4 s. (i) and (ii) in (A) and (B) represents two individual DONA spectra. Statistical distribution of vibrational bands corresponding to spin and oxidation state drawn from different single DONAs is shown in (C) and (D) for Au and Ag DONAs. (E) SERS spectra of hemin (153 μM) adsorbed on Au NPs (60 nm) surface (laser: 633 nm, objective: 50×, acquisition time: 10 s, laser power: 0.9 mW). (F) Contour plot of time evolution SERS spectra of hemin recorded from single Ag DONA (shown in inset; image size: 300 nm × 300 nm). Condition – laser: 488 nm; power: 80 μW; acquisition time: 0.5 s; objective: 100× (Zeiss). Temporal fluctuation in SERS intensity and spectral wandering in the vibrational modes characteristic of hemin can be seen of which most important are the *ν*_3_ (1497 cm^−1^) and *ν*_10_ (1611 cm^−1^) modes which signifies the high spin Fe(iii) state of hemin. White box highlights the peak wandering of *ν*_10_ band in the range of 1602–1616 cm^−1^ over time.

Next measurement step involved the addition of hemin (UV-vis spectra of hemin shown in Fig S11, ESI†) which undergoes complexation with G-quadruplex attached to the nanofork bridge *via* electrostatic interaction followed by dimerization to generate Au and Ag DONAs. Fig. 3A(i) shows SM-SERS spectra of the complex unit using Au DONAs (633 nm). A vibrational band attributed to asymmetrical stretching mode (C_α_C_m_) characteristic of the high spin configuration of hemin appears at 1606 cm^−1^ (*ν*_10_) along with a sharp band appearing at 1553 cm^−1^ (B_1g_) corresponding to *ν*_11_(C_β_–C_β_)_*asym*_ stretching of hemin. Excitation with 633 nm laser allowed preferential enhancement of the non-totally-symmetric mode *ν*_10_, although it is far off resonance with the Q-band.^37,54^ Other important vibrational bands corresponds to *ν*(C_α_N) stretching at 1157 cm^−1^ (*ν*_22_ with symmetry A_2g_) and *δ*(C_m_H) bending at 1253 cm^−1^ (*ν*_13_ with symmetry B_1g_; Fig. 3A(ii)) typical of type B or Hertzberg-Teller scattering.^36,37^ On the other hand, 488 nm excitation using Ag DONAs led to the classic type A scattering (Franck Condon) pattern additionally enhancing the totally symmetric mode *ν*_4_ belonging to A_1g_ symmetry. This is evident from the SM-SERS spectra that exhibit bands at 1371 cm^−1^ (Fig. 3B(ii)) along with peak at 1567 cm^−1^ (Fig. 3B(i)) due to *ν*_4_(Pyrrole half-ring)_*sym*_ and *ν*_2_(C_β_–C_β_)_*asym*_, which are absent and to most extent dampened respectively with 633 nm excitation.^36,37,55^ Band assignment for hemin can be found in Table S4, ESI.† ^45^ Although the laser excitation stands off resonance to the hemin Soret and Q-band, the SERS signal enhancement offered in the order of 10^9^–10^10^ by Au DONA and Ag DONA structures, provided the suitable condition for the SM characterization.^13^ Further, the absence of aromatic ring breathing band of hemin at 760 cm^−1^ in the SM-SERS spectra in Fig. 3A and B and other single DONAs analysed implies towards the preferential parallel orientation of the hemin plane to the nanoparticle surface in the hot-spot.

The vibrational modes *ν*_2_, *ν*_3_, and *ν*_10_ are typical signatures for the coordination and spin state of the hemin, whereas the *ν*_4_ vibrational mode acts as an indicator of the oxidation state of hemin.^35^ Typically, the SERS wavenumber for the ferrous (Fe(ii)) and that for the ferric (Fe(iii)) state lies in the range of 1354–1364 cm^−1^ and 1365–1372 cm^−1^, respectively. For example, the bands appearing at 1371 cm^−1^ (*ν*_4_; Fig. 3B(ii)) and 1567 cm^−1^ (*ν*_2_; Fig. 3B(i)) give a clear indication of hemin in Fe(iii) and five co-ordinated high spin state, respectively.^4,35,55^ To be noted, the *ν*_10_ band that corresponds to *ν*(C_α_C_m_)_*asym*_ stretch ideally appears at 1625 cm^−1^ for high spin ferric hemin (SERS assignment) when bound to nanoparticle surface (Fig. 3E). Interestingly, the same appears at around 1605–1616 cm^−1^ (Fig. 3A and B, in case of both Au and Ag DONAs, respectively) corresponding to the normal Raman wavenumber indicative of the unbound state of hemin.^50,55^ This indicates that a molecule need not necessary be bound to the plasmonic surface to show SM SERS signal. To further rule out the major contribution of bridge DNA, in the SM SERS spectra of G-quadruplex bound hemin, the SERS spectra of (GTT)_8_-SH and (TTT)_8_-SH coated Au NPs are shown as reference. Except for the 1450 cm^−1^ band which can be attributed to both hemin and bridge DNA, no significant interfering band was observed (Fig. S12A and B, ESI†). This is also supported by averaged SERS spectra obtained from multiple single Au and Ag DONAs, which show signals significant to hemin and G-quadruplex (Fig. S13A and B†). Importantly, no SM-SERS signal of hemin was observed from the single Au DONA structures without aptamer (Fig. S14(i), (ii) and (iv) ESI†) confirming the specificity of the aptamer-based approach for SM molecule detection (except as observed in Fig. S14(iii)†).

For further discussion on hemin chemistry we will focus mainly on the two characteristic marker bands *ν*_4_ and *ν*_10_ corresponding to spin and oxidation state. Statistical analysis of few SM-SERS spectra (histogram plot in Fig. 3C and D for both Au and Ag DONAs) shows the dominant population of high spin configuration of the G-quadruplex bound hemin in Fe(iii) state although the low spin configuration of hemin could also be observed, which remains obscured in ensemble spectra. The presence of a small number of molecules in low spin state could be attributed to (i) a strong binding of the EDTA (strong field ligand) present in 1× TAE/Mg^2+^ buffer to the Fe(iii) centre resulting in the population of low spin Fe(ii)/Fe(iii) hemin, or (ii) it could be that the hemin trapped at the hot-spot is in direct contact to the nanoparticle surface resulting in a shift of the wavenumber position. A small fraction of Fe(ii) state (Fig. 3D) could also be present due to charge tunnelling at the metal molecule interface.^35^ In the present experiment with Au DONAs (Fig. 3C) no detectable peak corresponding to *ν*_4_ vibrational mode (oxidation state indicator) could be observed, which can be ascribed to dormant symmetric mode excitation at 633 nm excitation.^36,37^

To further understand the SM behaviour, the time evolution SERS spectra from single Ag DONA was examined (Fig. 3F) by combined dark-field scattering microscopy and Raman time-series measurement (details in Methods section and Fig. S15†). Characteristic blinking pattern, peak wandering and relative fluctuation in intensity could clearly be observed in the SM-SERS time series for all the spectral peaks, although each of them exhibits different blinking behaviour at a single point of time.^56–58^ Interestingly additional new bands could be observed appearing in the range 1297–1311 cm^−1^, 1395–1409 cm^−1^ and 1527–1541 cm^−1^ corresponding to *δ*(C_m_H), *ν*_20_(B_2g_) and *ν*_38_(E_u_) modes of hemin (ESI Table S4†), which was not detected in correlated SM-SERS spectra reported above. The high spin state marker band *ν*_10_ clearly exhibits peak wandering in the range 1601–1619 cm^−1^ (indicated in white boxes) accompanied by intermittent blinking behaviour (Fig. 3E). The total peak disappearance (57 s–72 s) could be an indication that the hemin moiety flips out of the hot-spot volume due to thermal vibration. This is in tandem with blinking pattern observed for vibronically active bands (corresponds to non-totally symmetric mode) that typically shows erratic blinking behaviour with switch-on behaviour for brief time periods and off with weak intensity for longer period, however exhibiting the brightest intermittent signal while on.^55^ On the same line, totally symmetric modes guided by Franck–Condon interaction are steadier in intensity and show gradual drop-off or on behaviour with time.^56^ This is reflected from the *ν*_3_ band appearing in the range 1494–1505 cm^−1^ that remains steady over time with gradual disappearance and appearance observed in the initial time. The time-dependent fluctuation in SERS intensity (Fig. S16(i) and (ii),† typically shown for vibrational bands appearing at 1497 cm^−1^ and 1611 cm^−1^) reflects the blinking behaviour of the hemin at the hot-spot. Detailed discussion on the spectral behaviour in ESI.†

It is to be noted that the spin *ν*_10_ and oxidation state *ν*_4_ marker band and all other totally symmetric modes *ν*_2_, *ν*_3_ belonging to A_1g_ symmetry are most profoundly excited with Q- and Soret band excitation respectively. Although we are off-resonance, the 633 nm and 488 nm laser excitation used in the study (available laser source) still allows us to record SERS signal albeit weak. In this context, the inconsistencies in the peak should not be totally attributed to fluctuation freedom, conformational changes and thermal effects *etc*. however due to less overlap of laser excitation with the Q- and Soret band.

**Fig. 4:**
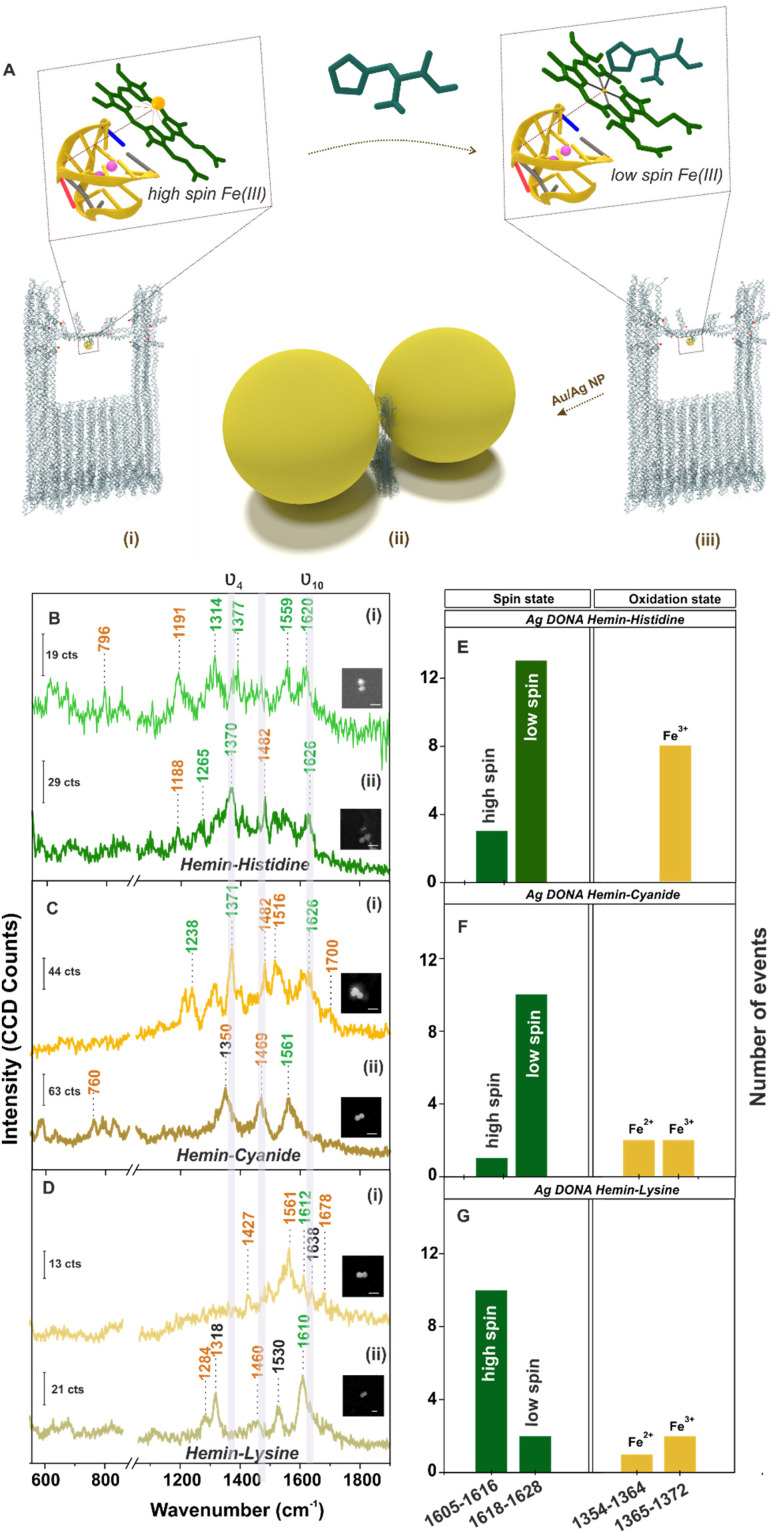
(A) Schematic representation of spin cross-over in hemin. Hemin bound to G-quadruplex in (i) high spin and (ii) low spin configuration – upon ligand binding (shown for histidine as an example) in the DNA origami nanofork bridge, (iii) nanoparticles (Au/Ag) coated with differently functionalised DNA sequence hybridised with complementary DNA capture strand (end labelled with red spheres) hanging out from the upper arms and bridge position of the DNA origami nanofork to form DONAs. Brown box indicates the plasmonic hot-spot of DONAs with G-quadruplex–hemin–ligand complex trapped therein. SERS spectra (inset shows SEM image of the corresponding DONAs; scale bar = 125 nm; measurement condition – laser: 488 nm; obj: 100×; power: 250 μW; acc. time: 4 s) of hemin bound Ag DONAs upon complexation with (B) histidine, (C) cyanide and (D) lysine and their corresponding statistical distribution of spin and oxidation state (E–G), respectively. (i) and (ii) in (B), (C) and (D) represents two individual DONA spectra.

Further, single molecule chemical interaction was investigated by monitoring the spin crossover in single hemin upon strong-field-ligand binding.^59^ For this, the G-quadruplex–hemin complex bound to the DNA origami nanofork is treated with three different ligands namely histidine, cyanide and lysine individually (refer Experimental section). Schematic depiction of the overall process is shown in Fig. 4A considering histidine as an example. The respective AFM images of the DNA origami nanofork (with G-quadruplex and hemin) treated with respective ligands are shown in Fig. S17, ESI.† Interestingly, the *ν*_10_ marker band appearing in the range 1605–1616 cm^−1^ in the SM-SERS showed a relative shift in wavenumber to the range 1618–1628 cm^−1^ in Fig. 4B(i), (ii) and C(i), (ii) upon histidine and cyanide binding, respectively, characteristic of low spin Fe(iii) hemin complex.^60,61^ The spin state switch is clearly reflected from the histogram plot (Fig. 4E and F) showing the statistical distribution of the *ν*_10_ and *ν*_4_ vibrational band extracted from single DONAs analyzed.

Unlike this, lysine addition interestingly did not induce any noticeable spin state change in hemin as reflected from the SM SERS spectra (Fig. 4D) as well from the statistical distribution in Fig. 4G that reflects dominant events corresponding to the high spin state. Plausibly, this can be attributed to the high p*K*_a_ value of the α- (9.0) and ε- (10.5) amino groups of lysine, which render it less nucleophilic to attack the Fe(iii) centre under the experimental condition of pH 8.0–8.4.^61^ This is well reflected from the average SERS spectra obtained from multiple single hemin bound Ag DONAs (Fig. S18, ESI†) treated with ligand. All band assignment remains the same as before. The same statistical plot drawn together considering the SM events as well as from ensembles of Ag DONAs also showed the same trend supporting the observed phenomenon (Fig. S19, ESI†). This therefore serves as a model study to use DONA structures to study physical properties, chemical interaction and transformation at a single molecule level using the principle of SERS.

## 4. Conclusions

Briefly, for the first time, we detect and investigate the molecular states and spin crossover of hemin *via* G-quadruplex mediated recognition by SM-SERS using plasmonic hot-spots offered by the DONAs (Au/Ag). Folding of the aptamer sequence held at the bridge position of the DONA to form the corresponding G-quadruplex in presence of K+ ions directs the hemin recognition. Owing to high field enhancement at the plasmonic hot spot, the detection of a single folded G-quadruplex and subsequently bound hemin becomes possible. Absence of the guanine ring breathing vibration (mostly) in successive SM-SERS of Au/Ag DONAs indicated towards the preferential orientation of the G-quadruplex plane parallel to the nanoparticle surface at the hot-spot. Flexibility offered by the DONAs structure to choose plasmonic nanostructures and hence the corresponding laser excitation source allowed excitation of the totally symmetric *ν*_4_ Raman modes of hemin (*i.e.*, using Ag DONAs and 488 nm excitation) which are absent/dampened with 633 nm laser excitation recorded with Au DONAs. Importantly, a systematic study by SM-SERS allows one to draw a statistical distribution of spin and oxidation states in hemin that remains screened in ensemble measurements and which indicates towards the preferential high spin Fe(iii) state of hemin. Importantly, the unique configuration of DONAs allows molecule to be trapped at the plasmonic hot-spot away from the metallic surface making room for native state molecular characterization. This is well reflected from the SERS spectra obtained in case of G-quadruplex and hemin (taking into account the conformational fluctuation) which otherwise is not possible in contact SERS at single molecule level.

Further, single molecule chemical interaction of hemin with strong field ligand (histidine, cyanide and lysine) is studied using Ag DONAs. Interestingly, a shift of the *ν*_10_ high spin marker band towards higher wavenumber characteristic of low spin confirmed the spin state switch upon ligand binding - showcasing the success of chemical interaction study. The present work therefore, opens up the possibilities of using such chemical moiety trapped in the hot-spot as Raman marker unit to monitor properties and dynamics of biologically Raman inactive molecules in addition to serve as DNA origami based plasmonic nanostructure platforms for aptamer-based detection of molecules relevant to diseases in single molecule scale.

## Author contributions

IB and AD conceived the idea and IB supervised the study; AD prepared and characterized the samples, did all the SERS measurements and data analysis and interpretation; KT helped in the SEM, TEM and time-dependent SERS measurements; AS, VC and GB performed MD simulations; all authors discussed the data and contributed to the manuscript writing.

## Conflicts of interest

The authors declare no conflict of interest.

## Supplementary Material

NR-014-D2NR03664A-s001
