# Supplementary material for: Molecular states and spin crossover of hemin studied by DNA origami enabled single-molecule surface-enhanced Raman scattering
Source: Nanoscale. 2022 Oct 28;14(44):16467–78. doi: 10.1039/d2nr03664a (PMC9671141; doi:10.1039/d2nr03664a)
Supplement: NR-014-D2NR03664A-s001 [file NR-014-D2NR03664A-s001.pdf]

Electronic Supplementary Material (ESI) for Nanoscale.
This journal is © The Royal Society of Chemistry 2022

Electronic Supporting Information

Molecular States and Spin Crossover of Hemin Studied by DNA Origami Enabled Single-Molecule Surface-Enhanced Raman Scattering

Anushree Dutta[a*], Kosti Tapio[a†], Antonio Suma[b], Amr Mostafa[a], Yuya Kanehira[a], Vincenzo Carnevale[c], Giovanni Bussi[d], Ilko Bald[a*]

[a]Institute of Chemistry, Physical Chemistry, University of Potsdam, Karl-Liebknecht-Str. 24-25, 14476, Potsdam, Germany.

[b]Dipartimento di Fisica, Università degli Studi di Bari, and INFN, Sezione di Bari, via Amendola 173, 70126 Bari, Italy

[c]Institute for Computational Molecular Science, Temple University, Philadelphia, PA 19122, USA

[d]Scuola Internazionale Superiore di Studi Avanzati, Via Bonomea 265, Trieste 34136, Italy

1. Atomistic simulations of the G-quadruplex

The atomistic structure of PS2.M was constructed following the proposed folding of ref 1. (monomeric with anti-parallel strands). The ideal model was then built using the 3D-NuS webserver.[2] Firstly, water [3] (TIP3P model) and ions[4] (0.1 M of KCl, parametrized by Joung and Cheatham) are added to the G-quadruplex (parametrized using the OL15 force-field).[5] The system is energy minimized using a steep descent algorithm. Afterwards, the system is equilibrated at a high temperature (400 K) for a short time (1 ns). We constraint, during equilibration, all the G-quadruplex hydrogen bonds, the torsional angle which impose planarity for the three quartet and the position of the K^+ ions between the quartet planes. The constraint are imposed using the PLUMED package[6] through harmonic restraints, which are subsequently lowered gradually during a second equilibration phase at 300 K for about 40 ns. The system was then simulated at 300 K for 1 μs. The software used for the simulation is GROMACS.[7] During both equilibration and simulations, we used periodic boundary conditions, constant pressure (1 bar) and long-range electrostatic interactions were treated by the particle mesh Ewald algorithm.[8] Configurations were sampled every 100 ps.

The groove width is measured as the backbone to backbone distance between the C4′ atoms of the central quartet (shown as blue spheres) in **Fig. 2A**, manuscript with label 1,2,3,4 corresponding to the C4′ atoms of G-nucleotides 4, 9, 13, and 18 using the sequence GTG GGT AGG GCG GGT TGG as reference for numbering). The average values are 1.46 nm, 1.35 nm, 1.47 nm, 1.38 nm, respectively for 1-2, 2-3, 3-4 and 4-1 C4′ distances (distributions and values as a function of time are in **Fig. S7A-B**). **Fig. S7C** shows the distribution of the vertical G-quadruplex length, defined as the maximum distance perpendicular to the G-quartet planes between all backbone atoms (see **Fig. 2A,** manuscript). The most probable value calculated for the vertical length is 1.99 nm, which is within the theoretical hot-spot gap distance in the DONAs.

2. Coarse-grained simulations of the nanofork - G-quadruplex complex.

The setup for the oxDNA2 coarse-grained simulation was prepared in the following way. The caDNAno template for the nanofork and the atomistic G-quadruplex DNA were converted to the oxDNA representation using the tacoxDNA package.[9] They were then merged and linked using oxView[10], and subsequently relaxed with the protocol described in ref 8. Simulations

were carried out with the LAMMPS software implementation.[11] The temperature was set to T= 300 K and the monovalent salt concentration to 1 M (typically large in order to have stable origami structures). Note that the G-quadruplex structure is not stable with the oxDNA model, due to the lack of Hoogsteen-Watson Crick pairing and of explicit counterions. Thus the G-quadruplex was constrained to its initial predicted position through a harmonic restraint on the root mean square deviation. The system was simulated for about 5×10^6 simulation time units, and configurations sampled every 10^3 time units.

In order to measure the orientation of the G-quadruplex planes with respect to the nanofork, we used the nanofork to define a fixed cartesian coordinate system (x-axis is the bridge, z-axis the pillars as represented), see the image below. This coordinate system is used to compute the relative orientation **P** of the G-quadruplex over the nanofork, where **P** is a normalized vector shown in the enlargement (red circle), connecting the lower to upper G-quartet centres and expressed in spherical coordinates (polar and azimuthal angles (θ,φ), while the radial distance is fixed to one). The histogram of (θ,φ) (not shown) is compatible with a random orientation of the **P** vector.

Table S1. List of modified DNA origami nanofork strand sequence.

Modified sequence name	Staple strand from ref 12	Modified sequence (red highlighted sequence corresponds to the PS2.M hemin aptamer)	5′-end	3′-end	Company
**S-161-PS2.M aptamer**	161	*GTG GGT AGG GCG GGT TGG TTT TAA AGC ACT AAA TCG GAA CCC TTG AAT CGG CCA ACG CGC GGG G*			Sigma Aldrich
**S-173 NP cap strand**	173	*AAA AAA AAA AAA AAA AAA AAA AAA AAAGGG AGC CCC CGA TTT AGA GCT TAAACC TGT CGT GCC AGC TGC ATT AAA ACAACA ACA ACA ACA ACA ACA AC*			Metabion
**S-129 NP capture strand**	129	*AAA AAA AAA AAA AAA AAA AAA AAA TTTTTG GGG TCG AGG TGC CGT AGA GGC GGT TTG CGT AAT GGG AAC AAC AAC AAC AAC AAC AAC AAC*			Metabion
**S-161-C343-aptamer-FAM**	161	*6-FAM(Fluorescein)-TGT GGG TAG GGC GGG TTG GT-Coumarin(343) –TTT TAA AGC ACT AAA TCG GAA CCC TTG AAT CGG CCA ACG CGC GGG G*	FAM		Metabion

Table S2. Spectral assignment of the SERS band observed with PS2.M aptamer *(GTGGGTAGGGCGGGTTGG)* in the DNA origami nanofork bridge of Au and Ag DONAs.

experimentally observed ῦ (cm^{-1}) in current study	SERS (cm^{-1}) literature	Raman wavenumber (cm^{-1})	Assignment	Reference
**1198 (Au)** **1195 (Ag)**	1180		Deoxythymidine (dT)	13
**1298 (Au)** **1278 (Ag)**	1275/1278		Ring stretching and C-H bending of thymine	13
**1497 (Ag)**		1496	C8=N7 when it is not H-bonded	13
**1589 (Au)**		1580/1582	Deoxyguanosine (dG) ring vibration	13,14
**1536 (Au)**	1536		Not assigned (from DNA origami nanofork bridge DNA sequence) **Fig. S11A**	
**1572 (Ag)**		1577	dG N2H deforнation (N2H interbase H bond)	15

Table S3. Spectral assignment of the SERS band observed after G-quadruplex formation in presence of K^+ for Au and Ag DONAs.

experimentally observed ῡ (cm^{-1}) in current study	SERS(cm^{-1}) literature	Raman wavenumber (cm^{-1})	Assignment	Reference
**640-660**			Guanine ring breathing	13
**682**			C2'-endo/anti deoxyguanosine	13
**833**	830		Sugar vibration C2′ -endo	13
**768/759**		748/750	OPO anti-sym	13
**1063**	1077		PO_2^- str	13,15
**1184**	1180	1180	Deoxythymidine (dT)	13
**1228/1236**		1240	dT, N-H def, C-N str	13
**1284/1285**	1278		Ring stretching and C-H bending of thymine	13
**1485/1482/1460/1464/1470**	1460/1480	1482/1480	C8=N7-H2 deformation	13,14
**1594/1591/1598**	1597/1598	1595	dG N1H hydrogen bond to water	15
**1374**	1370	1370	dT	13
**1586**	1586	1580/1582	dG ring vibration	13,15
**1395**	1396	1363	C2′ endo/anti dT	15
**1534**	1539		Purine ring stretch	14
**1522**	1510	1490	dG ring vibration (N7 hydrogen bond to water)	15
**1420**	1423/1424	1416	Deoxyribosyl ($C5'H_2$) def	13
**1634-1640**	1636		Ring stretching cytosine (C2=O)	16
**1676/1680/1698/1700**	1703	1670/1672	C6=O6-H1 stretch	13

Table S4. Spectral assignment of the SERS band observed for hemin in presence of K^+ for Au and Ag DONAs.

experimentally observed ῦ (cm^{-1}) in current study	SERS (cm^{-1}) literature	Raman wavenumber (cm^{-1})	Assignment	Reference
**710**			not assigned	
**1147/1157**		1124 (488 nm)<br>1120 (632 nm)	$\nu_{22}(C_\alpha N)$	17,18
**1253/1240**		1228	$\delta(C_m H)$ bending	18
**1340/1352**		1340 (488 nm)<br>1338 (632 nm)	ν_{41}(pyrrole-half ring)$_{str}$	18
**1371**	1354-1364 (Fe^{2+})<br>1365-1372 (Fe^{3+})	1341(514 nm)	ν_4(pyrrole-half ring)$_{sym\ str}$	18,19
**1450/1442**		1432 (488 nm)<br>1440 (632 nm)	$\nu_{40}(C_\alpha C_m)_{sym}$	18
		1492(488 nm)<br>1497(632 nm)	$\nu_3(C_\alpha C_m)_{sym}$	17,18
**1553**		1551(633 nm)	$\nu_{11}(C_\beta C_\beta)$	18
**1567**	1570 (514 nm)	1540 (514 nm)<br>1574(488 nm)<br>1570(632 nm)	$\nu_2(C_\beta C_\beta)$	18,19
**1606/1608 (Au)**<br>**1607/1616**<br>**1609/1612(Ag)**<br>**1620/1626**<br>**(Ag)**	1626 (633 nm)	1605-1616 (high spin)<br>1618-1628(low spin)	$\nu_{10}(C_\alpha C_m)_{asym}$	18,19

**1660**	1664		ν(C=O)$_{str}$ of surface bound carboxylic group	19
**1297-1311**		1307 (488 nm)<br>1309 (633 nm)	δ(C$_m$H)	18
**1395-1409**		1399/1398/1403	ν$_{29}$(pyrrole-quartet ring) or, ν$_{20}$	17,18
**1527-1541**		1553	ν$_{38}$(C$_β$C$_β$)	17,18

Discussion: The vibrational modes ν$_2$, ν$_3$, and ν$_{10}$, which correspond to ν(C$_β$C$_β$), ν(C$_α$C$_m$)$_{sym}$ and ν(C$_α$C$_m$)$_{asym}$ stretching, respectively, are typical signatures for the coordination and spin state of the hemin. Whereas, the ν$_4$ (A_{1g}) vibrational mode corresponding to $C-N$ stretch vibrations of pyrrole subunits (sensitive to electron transfer into π^* orbital of the porphyrin ring) serves as an indicator of the oxidation state of hemin. Typically, the SERS wavenumber for the ferrous (Fe(II)) and that for the ferric (Fe(III)) state lies in the range of 1354-1364 cm^{-1} and 1365-1372 cm^{-1}, respectively.[20]

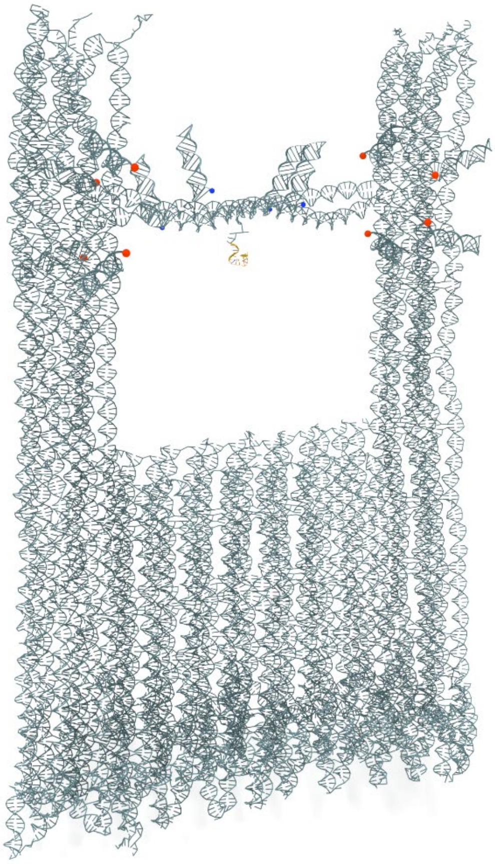

Fig. S1 Schematic view of the DNA origami nanofork structures showing the front view of the nanofork with PS2.M aptamer in the bridge (yellow DNA sequence). The bridge and arm capture sequence protruding from each side of the fork are shown in sequence ending with blue dots and red dots respectively.

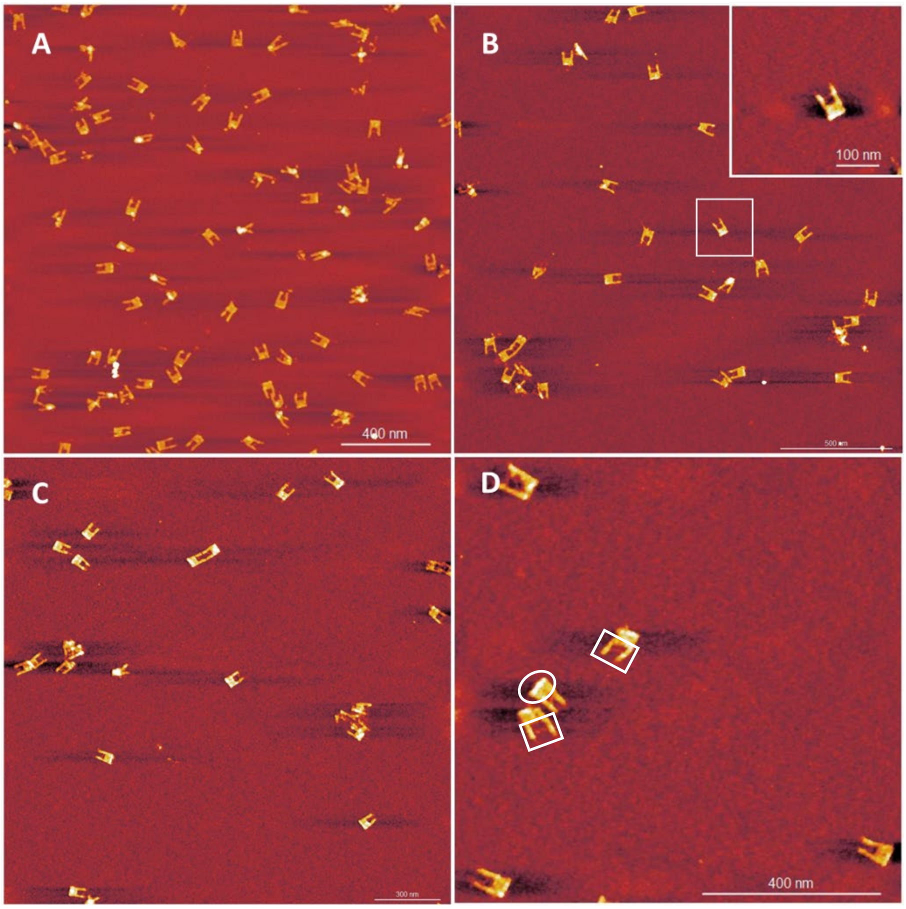

Fig. S2 (**A-C**) AFM images of DNA origami nanofork samples with hemin bound to the bridge. (**D**) White rectangular boxes highlights the double stranded running bridge sequence in the forks and white circle highlights the extra rows of DNA double helices imparting support to the base of the nanofork.

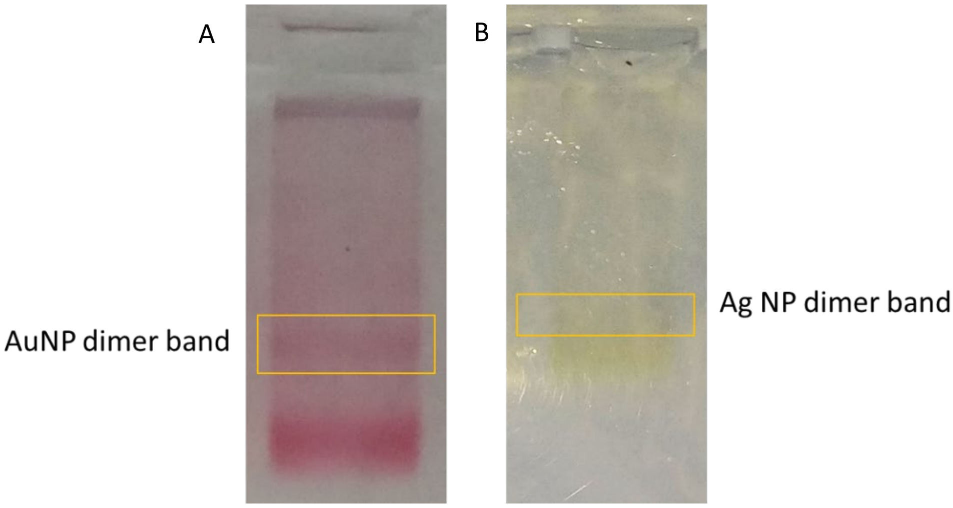

Fig. S3 Snapshot of (**A**) Au and (**B**) Ag DONAs sample after gel electrophoresis run. The yellow rectangular box indicates the Au and Ag DONA dimer band.

Representative AFM images of Au DONA Hemin

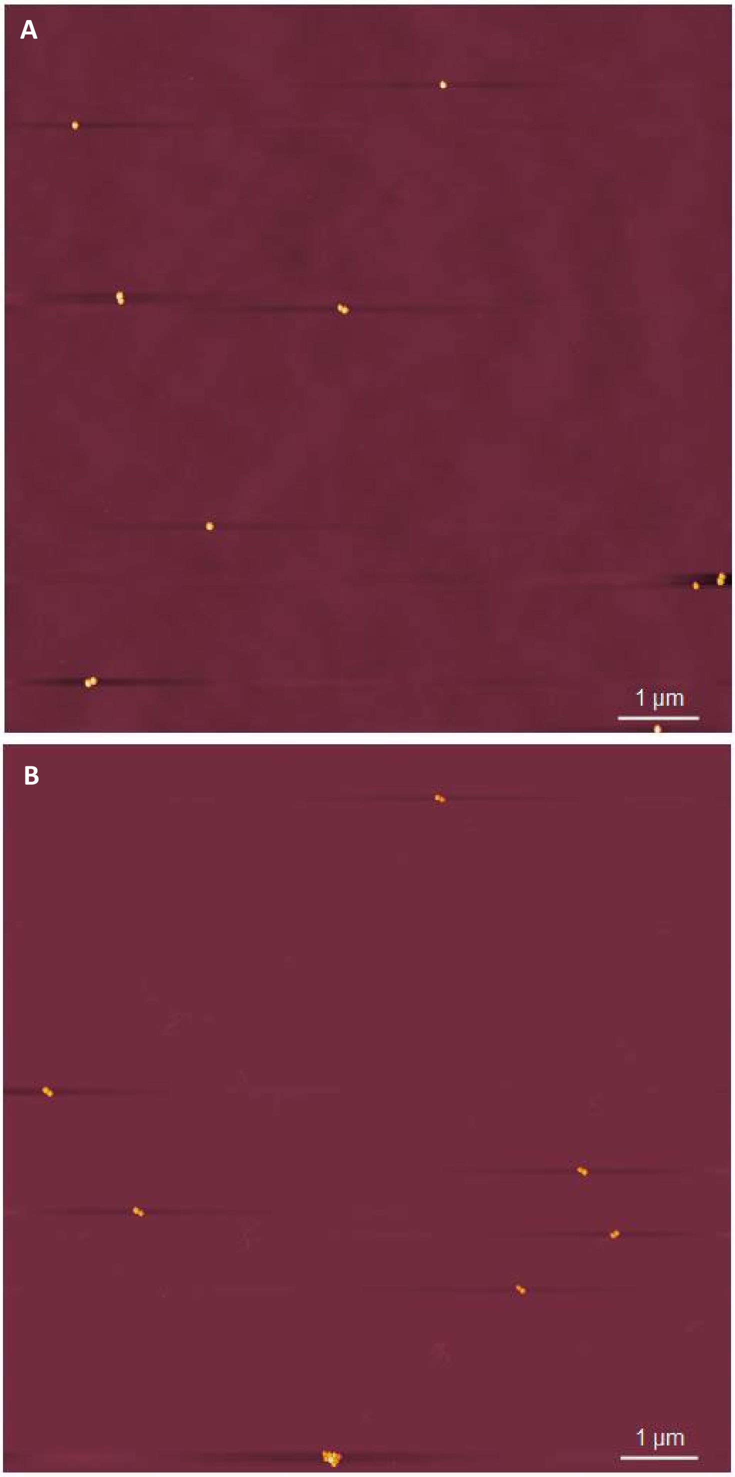

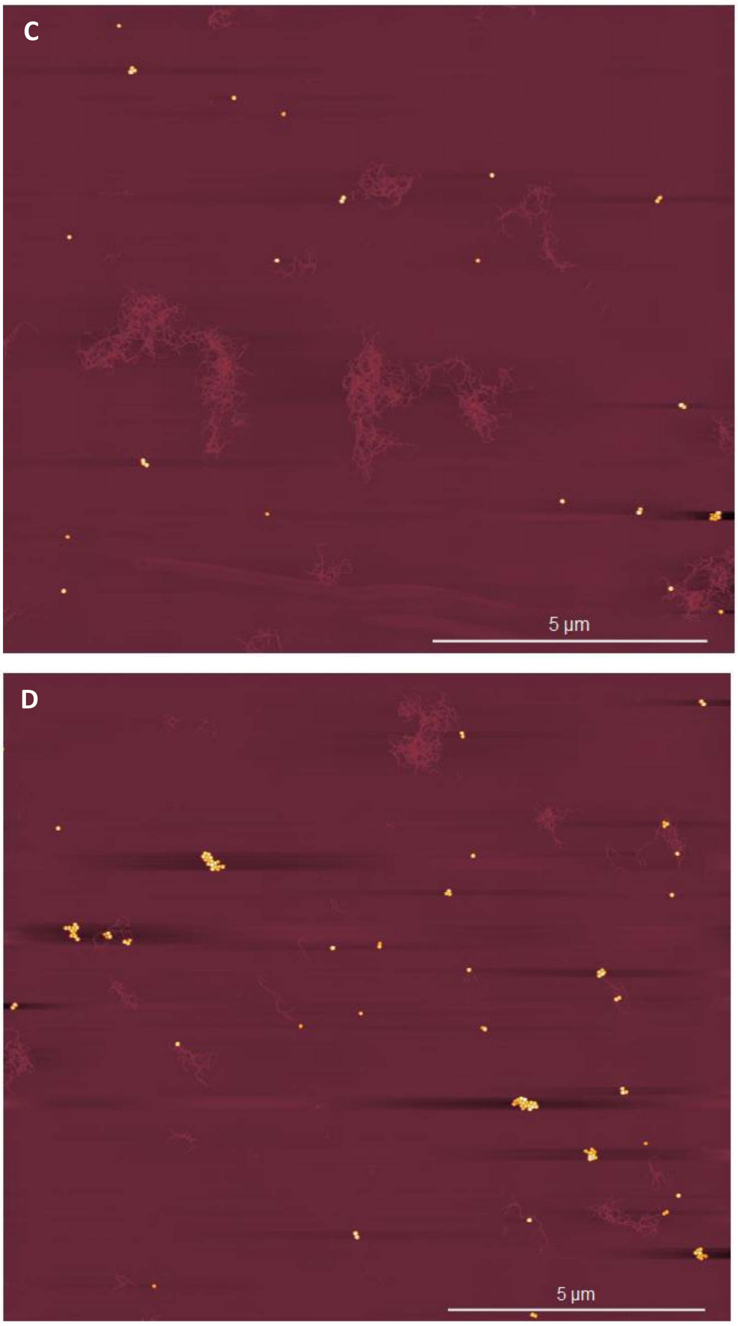

Fig. S4 (A-D) AFM images of as prepared hemin bound Au DONA sample deposited on Si substrate after agarose gel purification. Au DONA dimers highlighted in yellow circles.

AFM images of Ag DONA Hemin

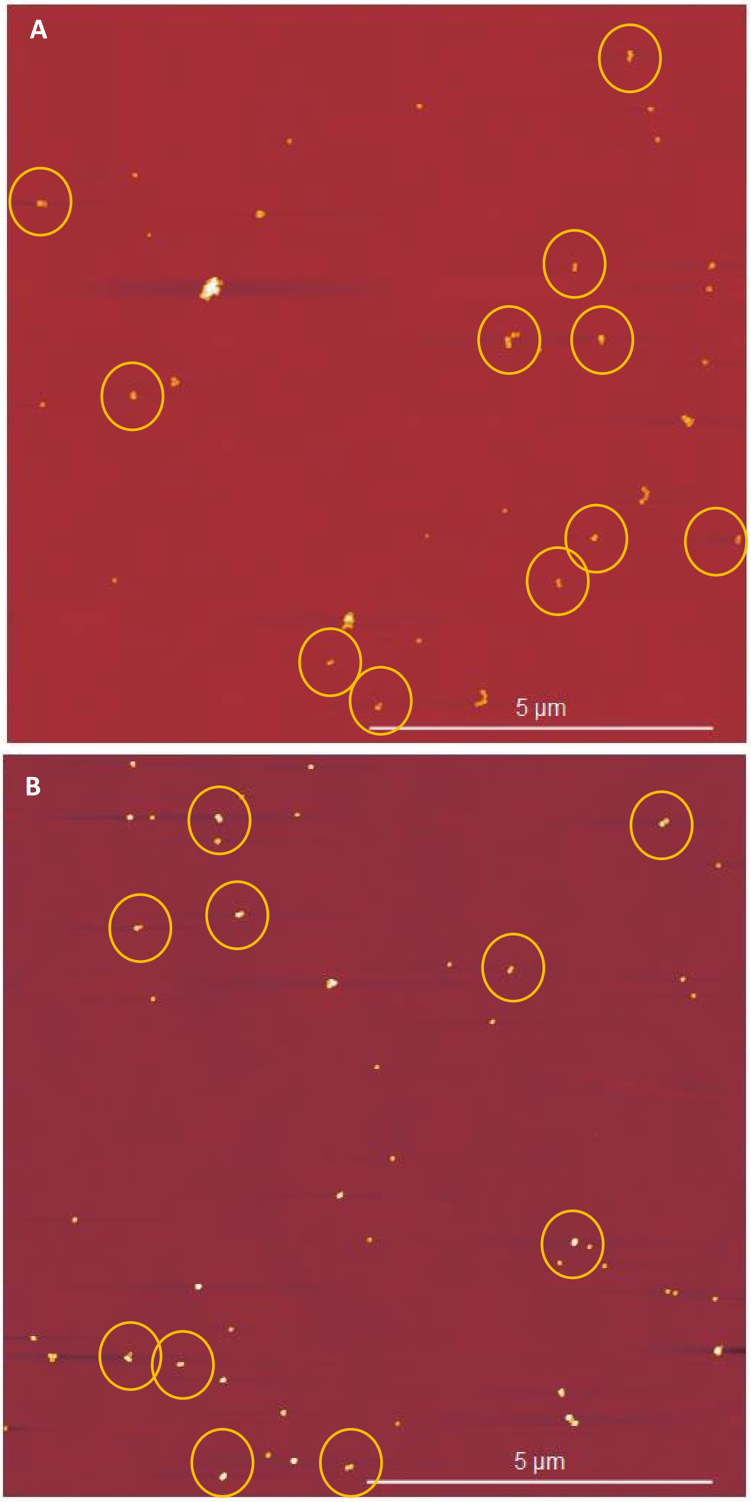

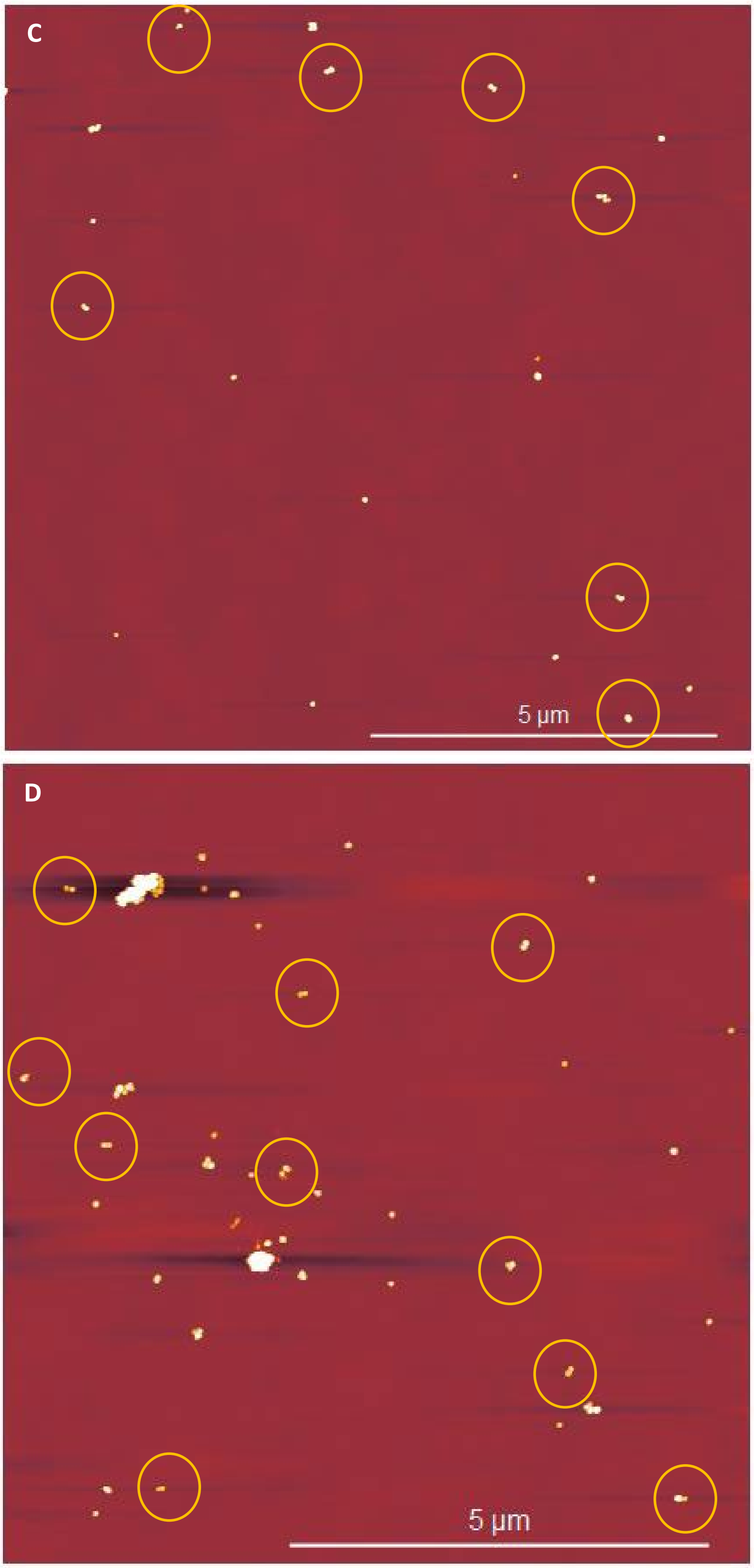

Fig. S5 (A-D) AFM images of as prepared hemin bound Ag DONA sample deposited on Si substrate after agarose gel purification. Ag DONA dimers highlighted in yellow circles.

3. FRET experiment to demonstrate the successful folding of hemin aptamer to G-quadruplex:

To ensure the conformational change induced G-quadruplex formation, we carried out a (Förster resonance energy transfer) FRET measurement employing coumarin (C343) and fluorescein (6-FAM) as donor-acceptor dye pair.[21] The aptamer sequence was modified with FAM dye at the 5′-end and C343 dye at the 3′-end, which is connected to the DNA origami fork via running bridge staple sequence separated by two T-spacer sequence. (**Fig. S6A and B**)

Emission spectra recorded for the aptamer sequence at the bridge of the DNA origami fork showed dominant emission spectra of C343 dye at 498 nm (**Fig. S6C**) before K^+ addition. Successful folding of the aptamer sequence upon addition of K^+ into a G-quadruplex leads to the close up pairing of the dyes resulting in efficient FRET as depicted in **Fig. S6B**. This is reflected in **Fig. S6C** (blue curve) with an additional shoulder appearing at 515 nm from FAM due to successful FRET.

A

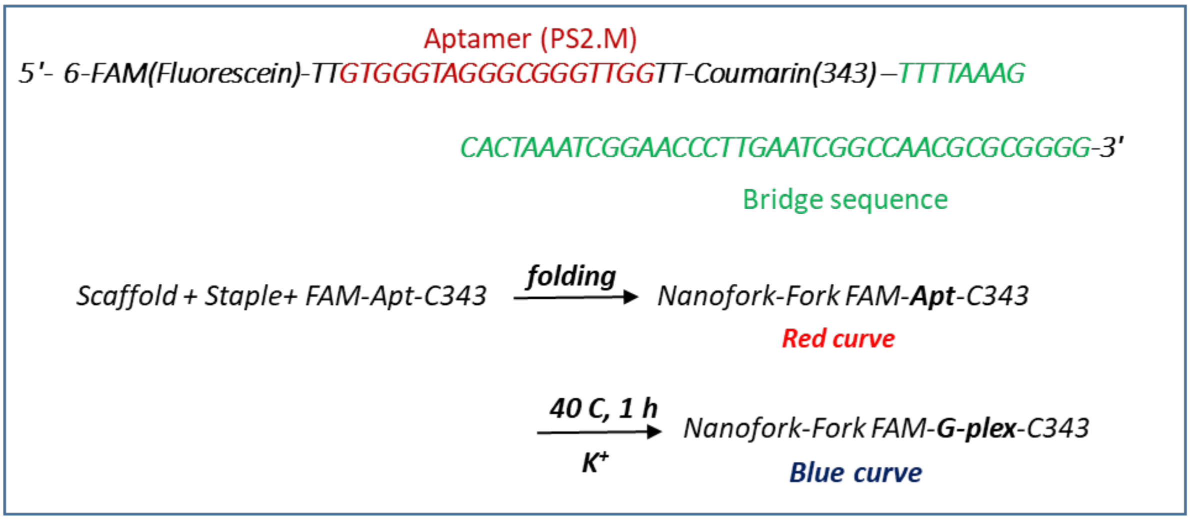

B

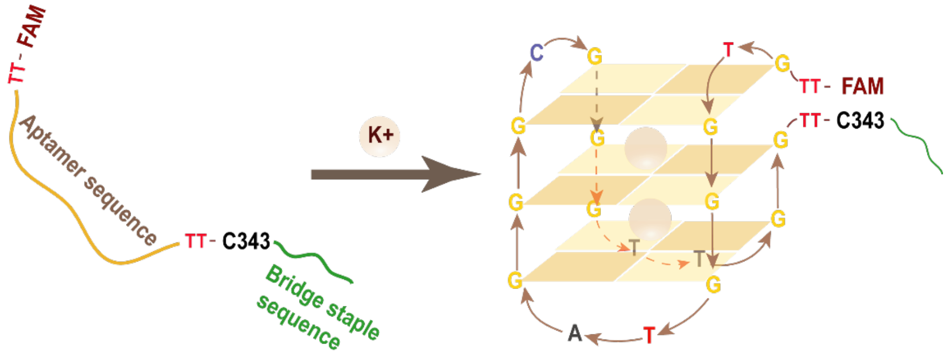

C

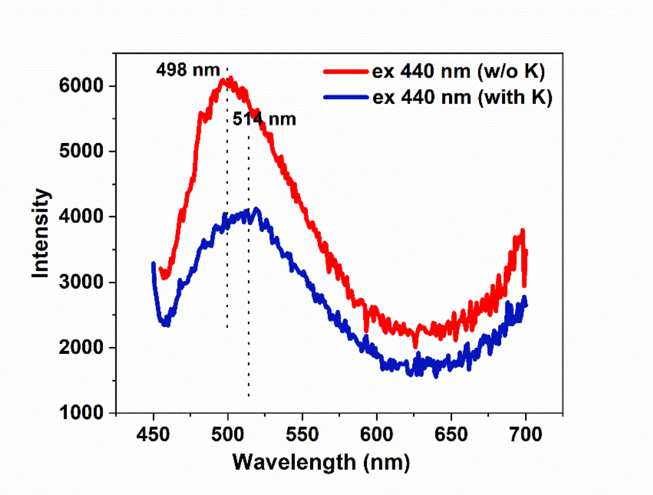

Fig. S6 (A) S161 bridge sequence appended to PS2.M aptamer with FRET dye pairs – coumarin-343 (C-343) and fluorescein (FAM), **(B)** folding steps of DNA origami nanofork with the FRET dye pairs leading to G-quadruplex formation in presence of K^+ ion, **(C)** FRET measurements showing the emission intensity versus wavelength plot of DNA origami nanofork before addition of K^+ (red curve) and that after the addition of K^+ (blue curve). Excitation wavelength = 440 nm

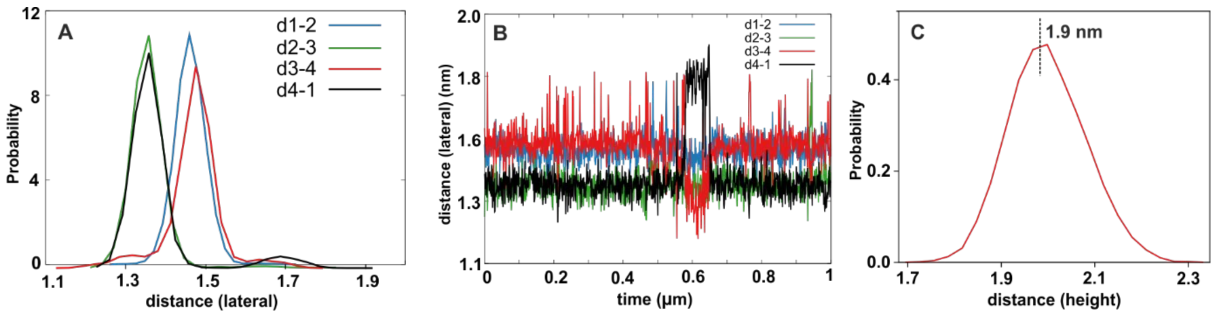

Fig. S7 (A) Groove widths computation for the central quartet considering the backbone to backbone distance between the four C4′ atoms numbered in blue spheres in **Fig. 2A**, see main

text and (**B**) corresponding plot of the groove width distance as function of time and (**C**) Distribution of the vertical distance in G-quadruplex with maxima around 1.9 nm.

Discussion: The average groove width measured considering the backbone to backbone distance are 1.46 nm, 1.35 nm, 1.47 nm, 1.38 nm, respectively for 1-2, 2-3, 3-4 and 4-1 C4′ (blue dots, **Fig. 2A;** manuscript) distances (distributions and values as a function of time are in **Fig. S7A-B**). On the other hand, the most probable value calculated for the vertical length of the G-quadruplex is 1.99 nm (**Fig. S7C**).

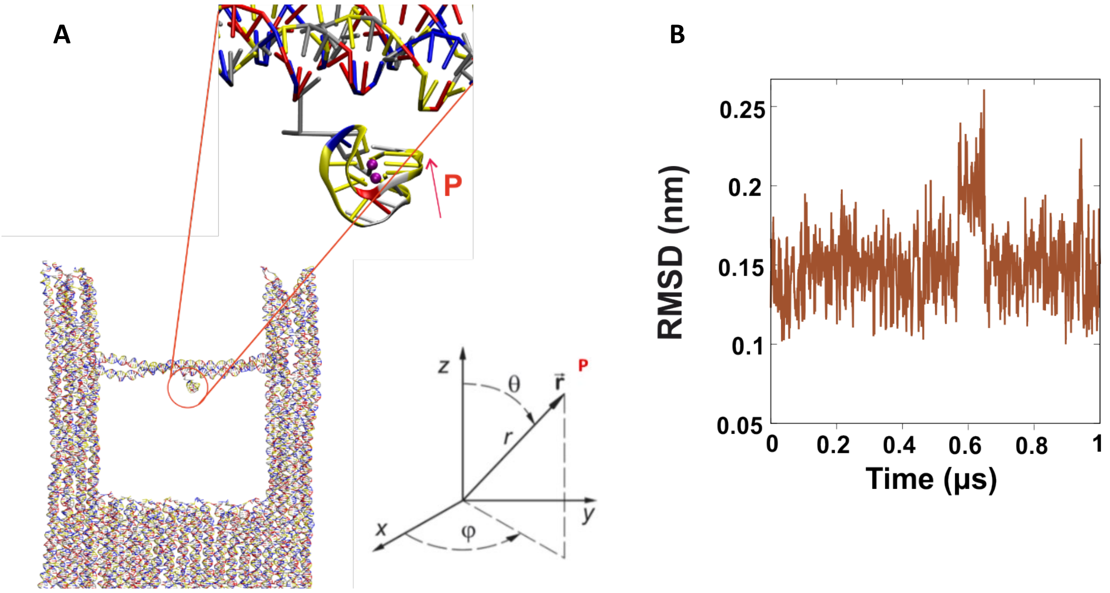

Fig. S8 (A) Snapshot of the G-quadruplex attached to the nanofork from the coarse-grained oxDNA2 molecular dynamics simulations. Magnified view of the simulated G-quadruplex at the bridge (red circle) showing one of the possible orientation in the bridge by defining a vector P (considering a spherical coordinate system) as shown in right. (B) Root mean square deviation (RMSD) with respect to ideal G-quadruplex as a function of time reflects the stable nature of the structure during the entire atomistic simulation.

Discussion: Based on the MD simulation, the G-quadruplex attached to the bridge can attain random orientation, but the absence (Figure 2E(i),(ii) and 2F(i), manuscript) or weak occurrence (Figure 2F (ii)) of the ring breathing mode 643 cm^{-1} indicates that there is a higher probability to adopt a parallel orientation to the nanoparticle surface.

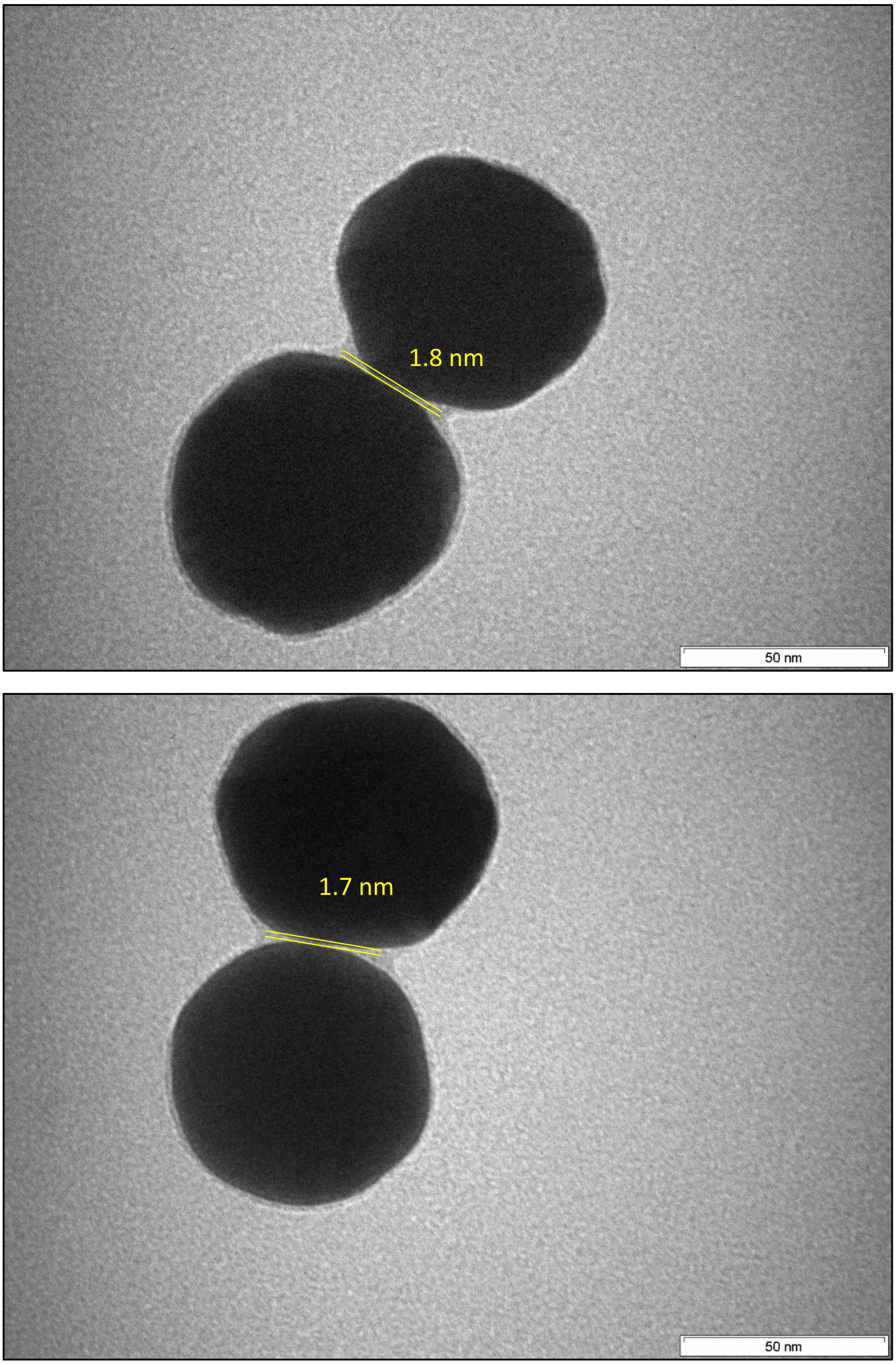

Fig. S9 Exemplary TEM images of Au DONAs. Because of the different material contrast the actual DONA structure could not be seen against nanoparticle although negative staining was used. The Au DONAs consists of 60 nm particles size. The gap distance calculated in each of the dimers are highlighted in yellow text. The average gap distance was calculated to be 1.7±0.13 nm. To be mentioned that DONAs with gap distance in the range of 1.1-1.3 nm were also found which were not taken into account in the statistical analysis. We infer such DONAs to be devoid of the G-quadruplex with hemin moiety.

Correlated Raman SEM map of Au DONAs (with hemin complexed to G-quadruplex in the center of the bridge

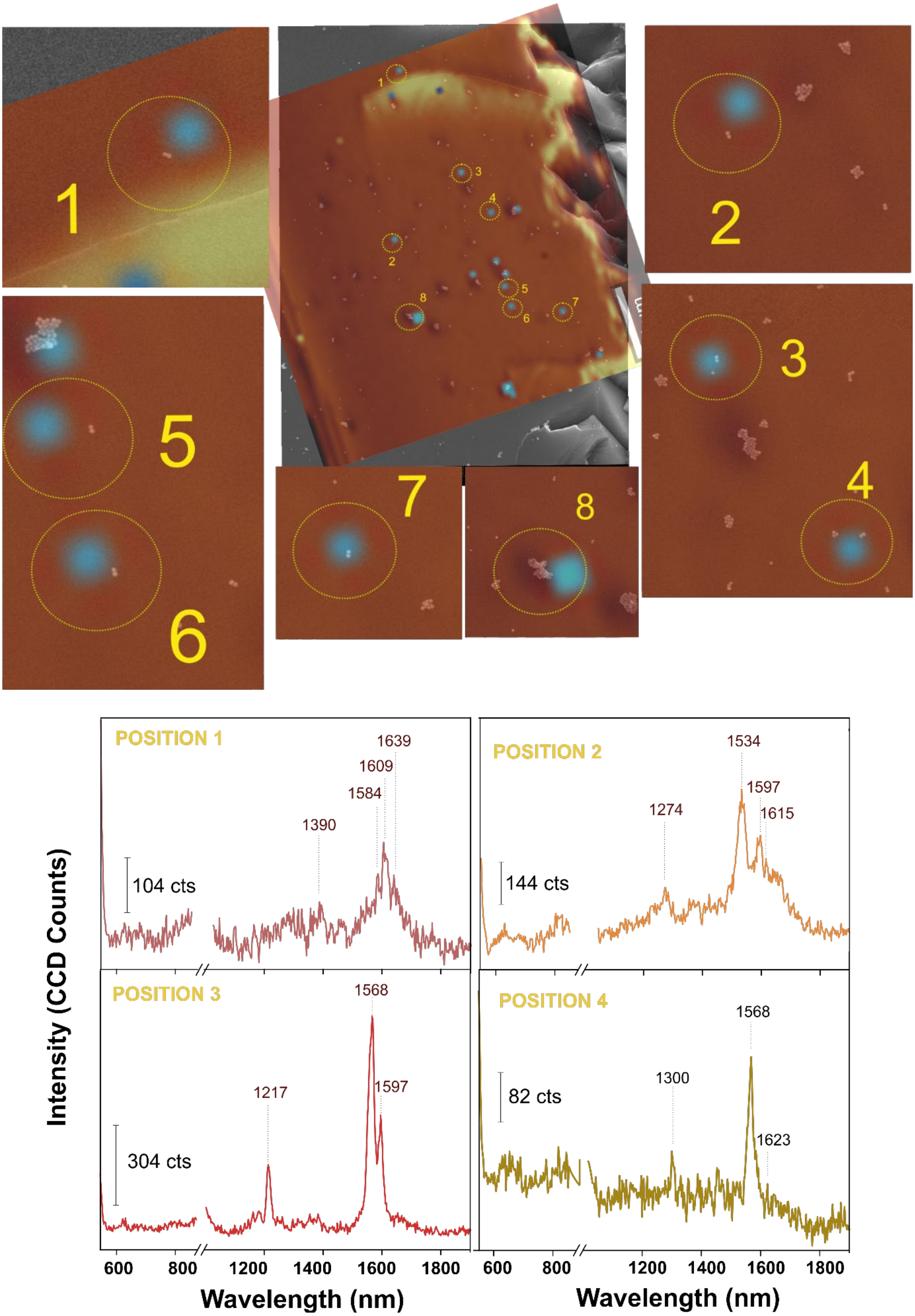

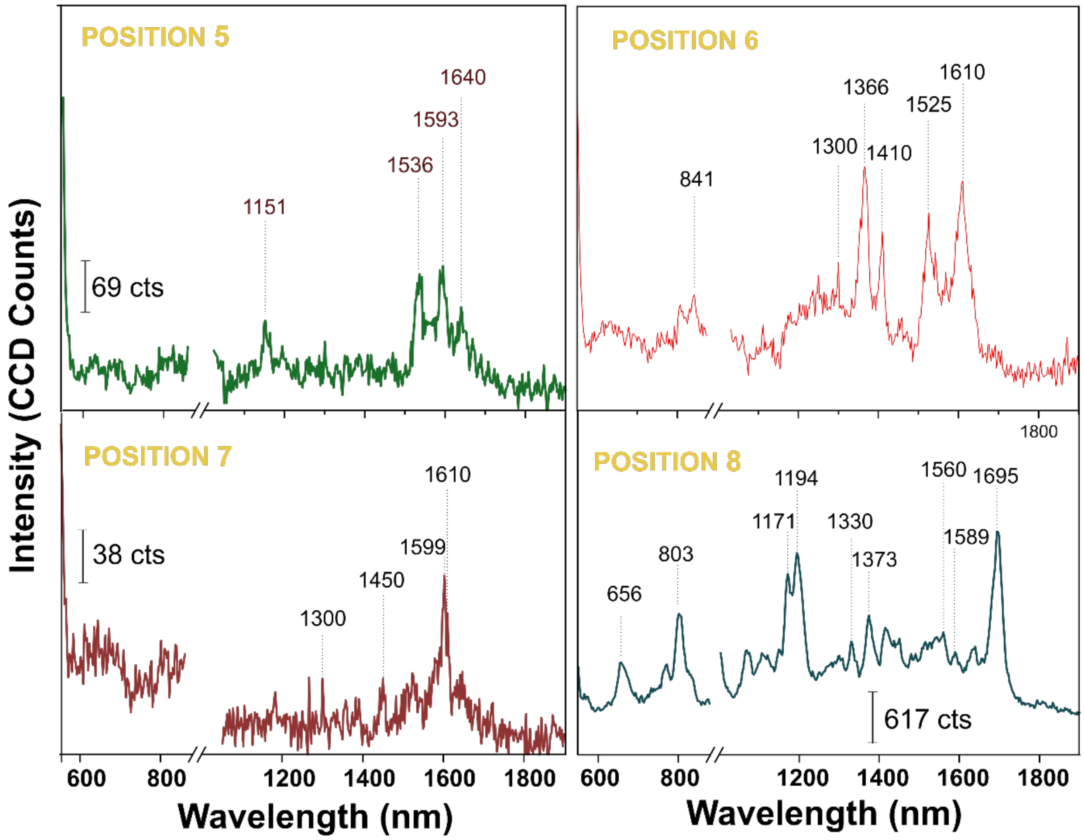

Fig. S10 (**A**) Correlated Raman SEM map of Au DONAs (with hemin complexed to G-quadruplex in the center of the bridge). Some selected single Au DONAs and aggregates of DONAs are circled in yellow. (**B**) Corresponding SERS spectra of single DONAs and aggregates that are circled and numbered respectively. Not all selected dimers showed the spin state marker band in the range ~1605-1618 cm^{-1}. The spectra corresponding to POSITION 8 represents aggregated DONAs. **Note:** Although POSITION 3 refers to single Au DONAs, however, the nearby aggregate could also contribute to the SERS signal. cts refers to CCD counts.

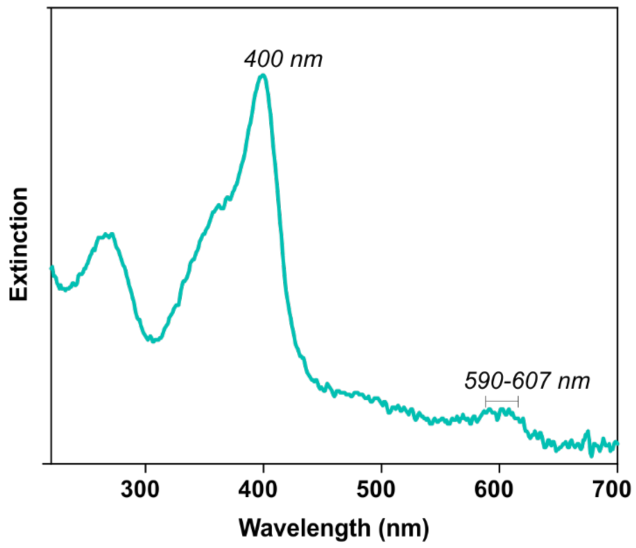

Fig. S11 UV-vis absorption spectra of 10 μM hemin solution (water-ethanol mixture). The UV-vis absorption spectra shows a main band at 400 nm and a broad band at around 590-607 nm that corresponds to the Soret band (B-band) and Q-band respectively which arises due to π- π* transitions of the porphyrin ring modulated by the ferric ion.

Discussion: The preferable interaction of hemin with the terminal end of the G-quadruplex is well documented in the literature and is attributed to the electrostatic attraction between positively charged Fe^{3+} and negative charges of oxygen in phosphate of sugar-phosphate backbone.[22]

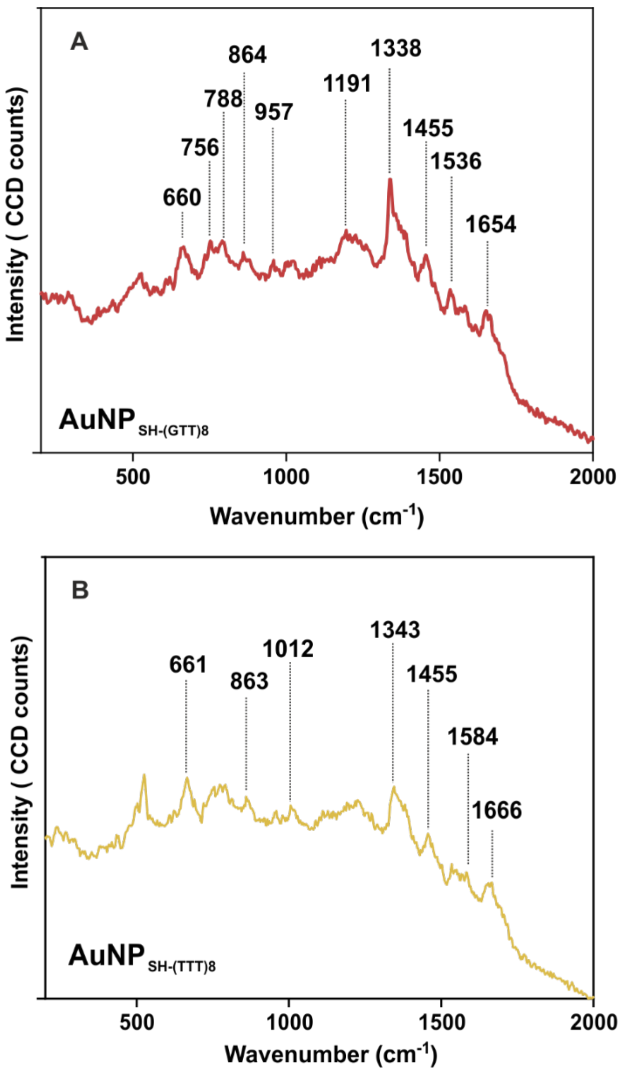

Fig. S12 SERS spectra recorded from (**A**) (GTT)8-SH and (**B**) (TTT)8-SH coated Au NPs deposited on Si-wafer with 633 nm laser excitation source. (10 s, acc 20, 50×, 250 μW)

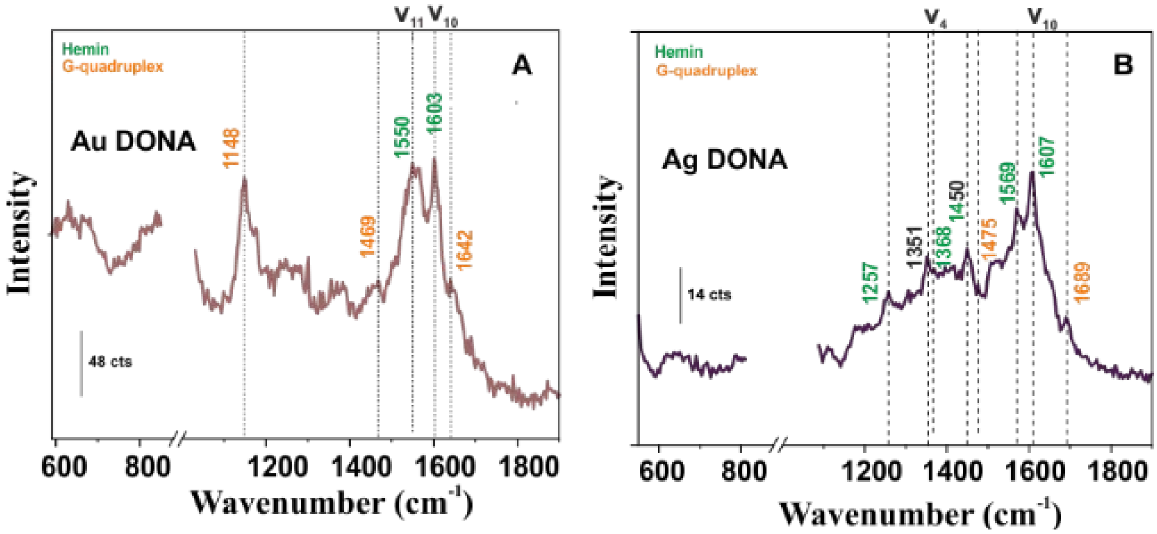

Fig. S13 Average spectra of multiple single (A) Au and (B) Ag DONAs.

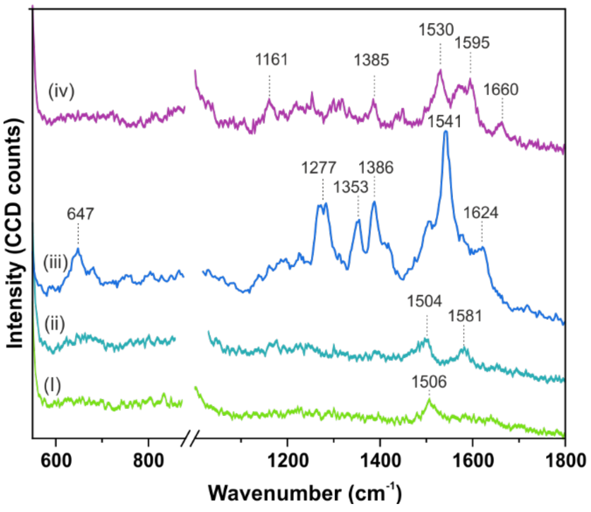

Fig. S14 Representative SERS spectra from Au DONA sample treated with hemin without PS2.M aptamer in the bridge. (i), (ii), (iv) represents single and (iii) aggregate Au DONAs.

In few situations, SERS signal was observed in Au aggregates (an exemplary situation shown in Fig S13 (iii)). This could arise if hemin remains intercalated to the nanofork body due to inefficient washing step, which might produce some SERS signal from aggregated dimer body.

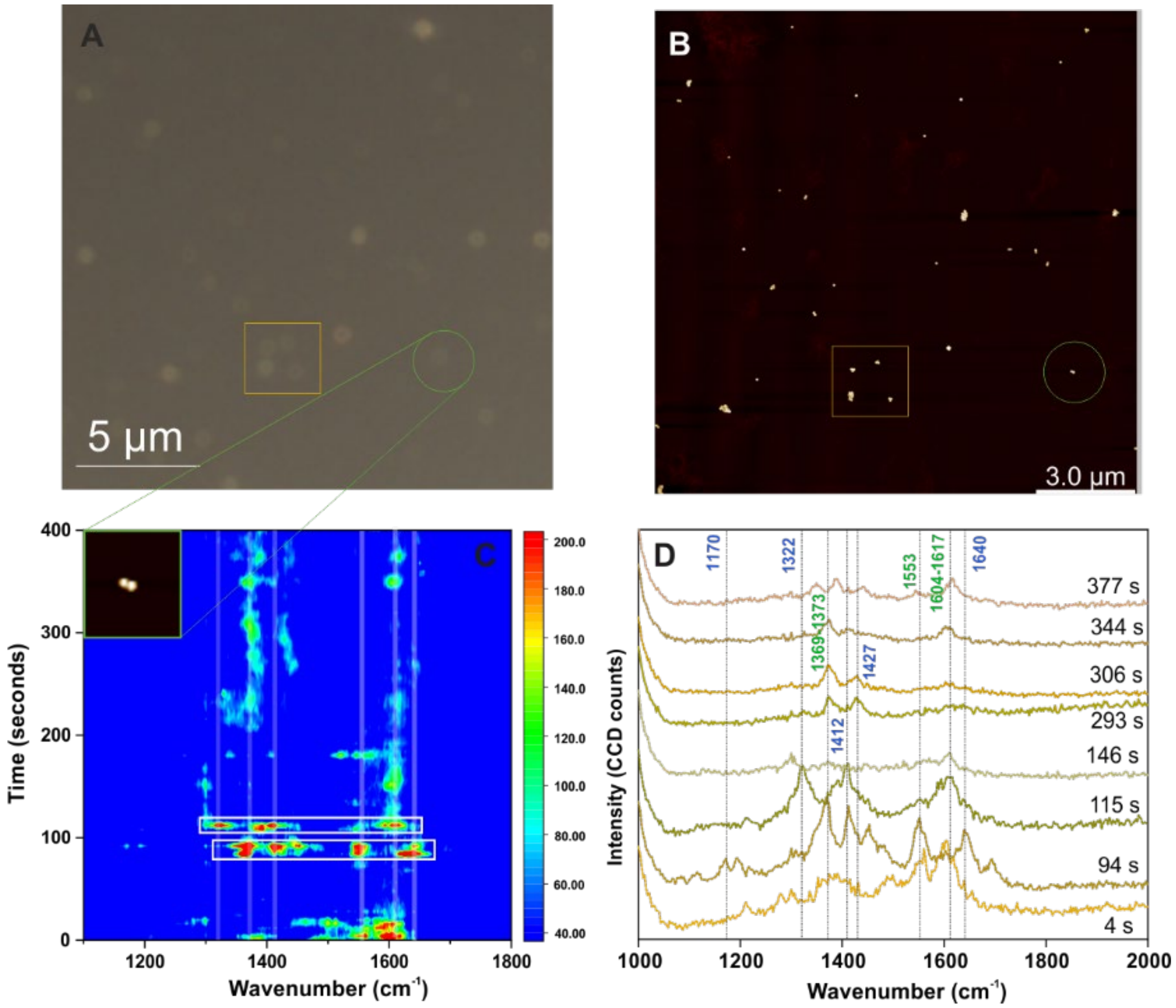

Fig. S15 Correlated DF and AFM images of Au DONAs and time series SERS spectra of a selected Au DONA. (**A**) DF scattering image of Au DONAs and the corresponding AFM image view of the same in (B). The area marked in yellow box was used as reference to spot dimers in the nearby area. A selected Au DONA is marked in green circle in (**B**) and the corresponding time series SERS measurement is shown in (**C**) as time versus wavenumber contour plot. Condition - laser: 633 nm; acquisition time: 4 s; measurement: 500; objective: 100x (Zeiss). The peak appearing at around 1322 cm^{-1} corresponds to G-quadruplex. Time series SERS so obtained showed spectral wandering at times highlighted in white box along with distinct blinking pattern – characteristic of typical single molecule behavior. (**D**) SERS spectra of single Au DONA extracted at different time interval from the contour plot. The wavenumber marked in blue represents peak from DNA and G-quadruplex and the one in green corresponds to hemin molecule.

Time series measurement on single Ag DONAs in dark field mode

To further understand the SM behaviour, the time evolution SERS spectra from single Ag DONA was examined (**Fig. 3E**) by combined dark-field scattering microscopy and Raman time-series measurement. (Refer experimental section for details and **Fig. S12,** ESI for time evolution SERS spectra from Au DONAs) Characteristic blinking pattern, peak wandering and relative fluctuation in intensity could clearly be observed in the SM-SERS time series for all the spectral peaks (although each of them exhibits different blinking behaviour at a single point of time) which could be attributed to molecular motion and its probable interaction with the substrate at the hot-spot, conformational changes, and/or thermal effects.[23-25] Interestingly additional new bands could be observed appearing in the range 1297-1311 cm^{-1}, 1395-1409 cm^{-1} and 1527-1541 cm^{-1} corresponding to $\delta(C_mH)$, $\nu_{20}(B_{2g})$ and $\nu_{38}(E_u)$ modes of hemin (**Table S4**, ESI), which was not detected in correlated SM-SERS spectra reported above. The characteristic peak of G-quadruplex around 1476 cm^{-1} could be observed during initial 18s which however remains off for prolonged period (**Figure 3E**) The high spin state marker band ν_{10} clearly exhibits peak wandering in the range 1602-1616 cm^{-1} (indicated in white boxes; **Fig. 3E**) accompanied by intermittent blinking behaviour. The behaviour includes shorter bright on-period (53 s-56 s) followed by total peak disappearance (57 s -72 s) which then gradually shows up as weak peak intensity for prolonged time to finally reappear as bright signal at 85 s to 97 s of the acquisition time (acquisition time was 0.5s). The total peak disappearance could be an indication that the hemin moiety could flip out of the hot-spot volume due to thermal vibration. This is in tandem with blinking pattern observed for vibronically active bands (corresponds to non-totally symmetric mode) that typically shows erratic blinking behaviour with switch-on behaviour for brief time periods and off with weak intensity for longer period, however exhibiting the brightest intermittent signal while on.[23] On the same line, totally symmetric modes guided by Franck-Condon interaction are steadier in intensity and show gradual drop-off or on behaviour with time[23] reflected from the ν_3 band appearing in the range 1494-1505 cm^{-1} that remains steady over time with gradual disappearance and appearance of the ν_3 band observed in the initial time. The time-dependent fluctuation in SERS intensity (**Fig. 3F (i)** and **(ii)**) typically shown for vibrational bands appearing at 1497 cm^{-1} and 1611 cm^{-1}, respectively, reflects the blinking behaviour of the hemin at the hot-spot. For example, the band at 1611 cm^{-1} shows an off-state during 20-50 s (**Fig. 3F(ii)**) of the time series measurement followed by brief sharp on-period for 6 s leading back again to off state during 60-70 s.

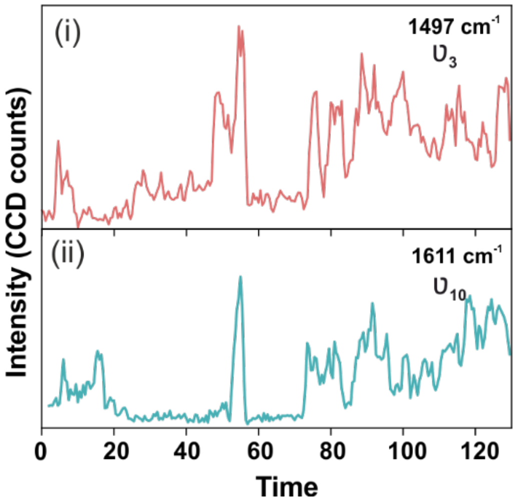

Fig. S16 Time-profile of fluctuation in SERS intensity (blinking) of band at (i) ~ 1497 cm^{-1} and (ii) 1611 cm^{-1} extracted from Fig. 3E.

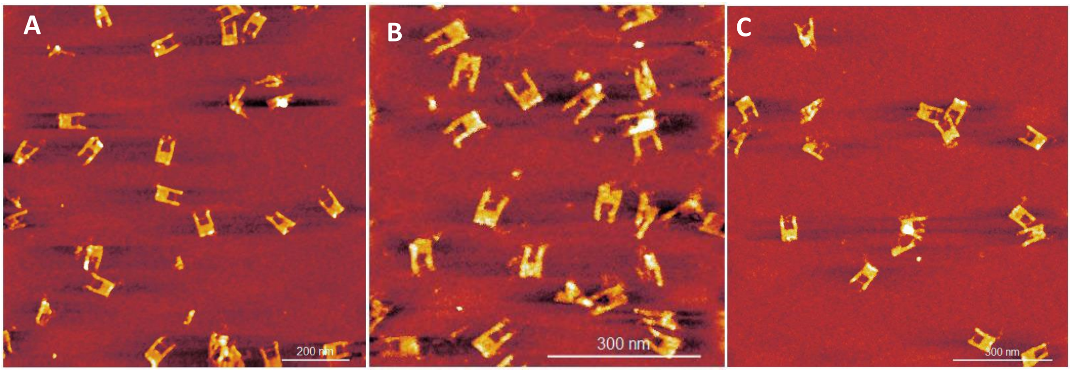

Fig. S17 AFM images of (A) histidine (B) cyanide and (C) lysine bound to the bridge of DNA origami nanofork.

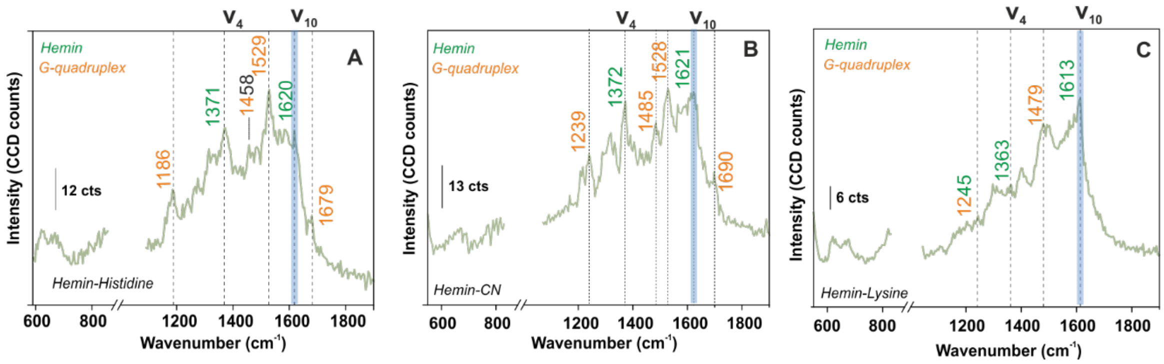

Fig. S18 Average SERS spectra from multiple Ag DONAs (hemin bound) upon complexation with (A) histidine, (B) cyanide (CN) and (C) lysine.

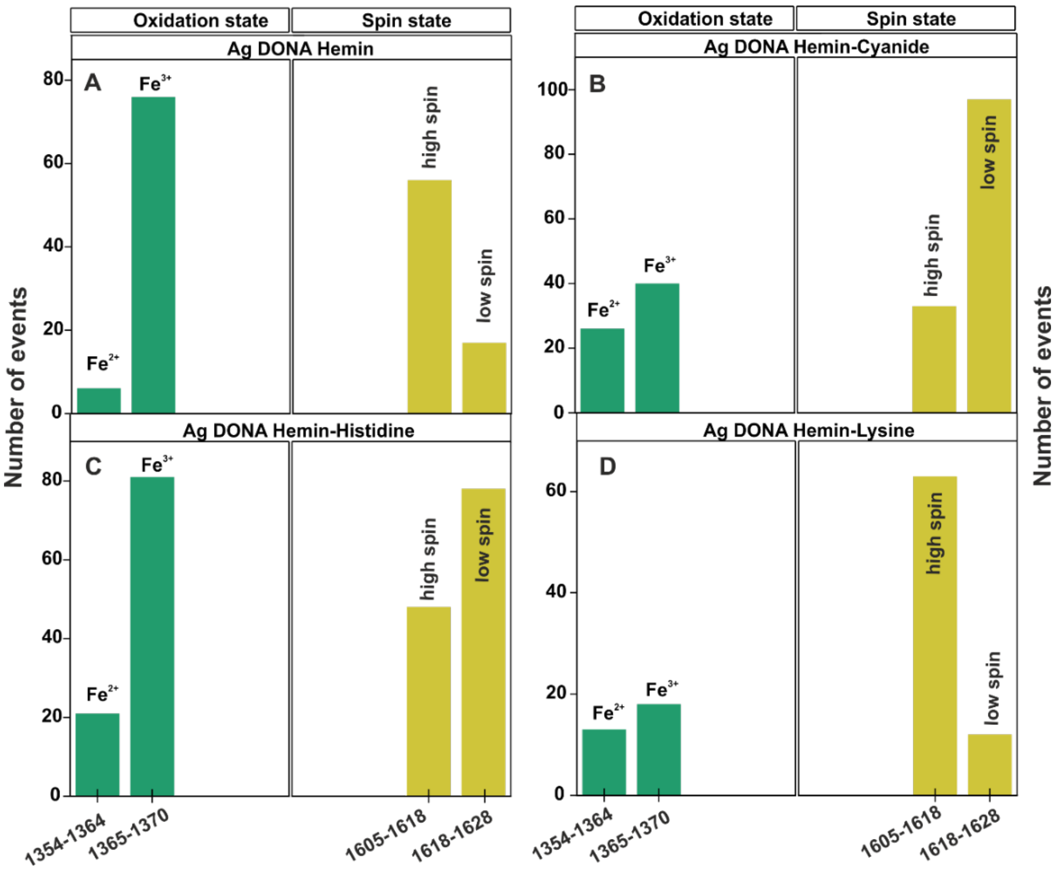

Fig. S19 Statistical distribution of spin and oxidation state from (**A**) hemin bound Ag DONAs and upon complexation of hemin with (**B**) cyanide, (**C**) histidine and (**D**) lysine. The distribution is an outcome of ensemble measurements considering dimers, trimers and n-mers.

Discussion: Although hemin ideally should be in a non-contact configuration in the plasmonic hot-spot, the fluctuation interaction (mentioned above) might result in redox state self-exchange and hence the observed distribution of Fe (II)/Fe (III) supported by a study by Wang et al.[20] where the fluctuation trajectories observed for ν_4 mode were ascribed to charge transfer dynamics that exist on Ag-hemin interface. Further they report that under no potential applied the single molecule of hemin tends to fluctuate between oxidized and reduced state passing through possible intermediate states consequently giving rise to one dominant distribution.

Ligand binding: The hemin Fe (III) unit which predominantly exists in high spin state should ideally switch to low spin Fe (III) state upon successful binding of the respective strong field ligands, resulting in a shift of the ν_{10} mode.[26-27]

References

1 Y. He, Y. Tian, C. Mao, H *Mol. Biosyst.* 2009, **5**, 238.

2 L. P. P. Patro, A. Kumar, N. Kolimi, T. Rathinavelan, *J. Mol. Biol.* 2017, **429**, 2438.

3 W. L. Jorgensen, J. Chandrasekhar, J. D. Madura, R. W. Impey, M. L. Klein, *J. Chem. Phys.* 1983, **79**, 926.

4 I. S. Joung, T. E. Cheatham, *J. Phys. Chem. B* 2008, **112**, 9020.

5 M. Zgarbová, J. Šponer, M. Otyepka, III T. E. Cheatham, R. Galindo-Murillo, P. Jurečka, *Chem. Theory Comput.* 2015, **11**, 5723.

6 G. A. Tribello, M. Bonomi, D. Branduardi, C. Camilloni, G. Bussi, G. *Comput. Phys. Commun. Comput. Phys. Commun.* 2014, **185**, 604.

7 M. J. Abraham, T. Murtola, R. Schulz, S. Páll, J. C. Smith, B. Hess, E. Lindah, *SoftwareX* 2015, **1–2**, 19.

8 U. Essmann, L. Perera, M. L. Berkowitz, T. Darden, H. Lee, L. G. Pedersen, *J. Chem. Phys.* 1995, **103**, 8577.

9 A. Suma, E. Poppleton, M. Matthies, P. Šulc, F. Romano, A. A. Louis, J. P. K. Doye, C. Micheletti, L. Rovigatti, *J. Comput. Chem.* 2019, **40**, 2586.

10 E. Poppleton, J. Bohlin, M. Matthies, S. Sharma, F. Zhang, P. Šulc, *Nucleic Acids Res.* 2020, **48**, E72.

11 O. Henrich, Y. A. Gutiérrez Fosado, T. Curk, T. E. Ouldridge, *Eur. Phys. J. E 2018,* **41***, 57.*

12 K. Tapio, A. Mostafa, Y. Kanehira, A. Suma, A. Dutta, I. Bald, *ACS Nano* 2021, **15**, 7065.

13 C. V. Pagba, S. M. Lane, S. Wachsmann-Hogiu, *J. Raman Spectrosc.* 2010, **41**, 241.

14 G. Rusciano, A. C. De Luca, G. Pesce, A. Sasso, G. Oliviero, J. Amato, N. Borbone, S. Derrico, V. Piccialli, G. Piccialli, L. Mayol, *Anal. Chem.* 2011, **83**, 6849.

15 Y. Li, X. Han, S. Zhou, Y.Yan, X. Xiang, B. Zhao, X. Guo, *J. Phys. Chem. Lett.* 2018, **9**, 3245.

16 F. Madzharova, Z. Heiner, M. Gühlke, J. Kneipp, *J. Phys. Chem. C* 2016, **120**, 15415.

17 B. S. Yeo, S. Mädler, T. Schmid, W. Zhang, R. Zenobi, *J. Phys. Chem. C* 2008, **112**, 4867.

18 B. R. Wood, S. J. Langford, B. M. Cooke, J. Lim, F. K. Glenister, M. Duriska, J. K. Unthank, D. McNaughton, *J. Am. Chem. Soc.* 2004, **126**, 9233.

19 R. T. Tom, T. Pradeep, *Langmuir* 2005, **21**, 11896.

20. Y. Wang, P. C. Sevinc, Y. He, H. P. Lu, *J. Am. Chem. Soc.* 2011, **133**, 6989.

21. L. Olejko, P. J. Cywinski, I. Bald, *Angew. Chemie - Int. Ed.* 2015, **54**, 673.

22 M. Ghahremani Nasab, L. Hassani, S. Mohammadi Nejad, D. Norouzi, *J. Biol. Phys.* 2017, **43**, 5.

23. J. R. Lombardi, R. L. Birke, G. Haran, *J. Phys. Chem. C* 2011, **115**, 4540.

24. Y. Maruyama, M. Ishikawa, M. Futamata, *J. Phys. Chem. B* 2004, **108**, 673.

25. S. R. Emory, R. A. Jensen, T. Wenda, M. Han, S. Nie, *Faraday Discuss.* 2006, **132**, 249.

26. N. Nanzyo, S. Sano, J. Biol. Chem. 1968, 243, 3431.

27. H. Wilgus, E. Stellwagen, Proc. Natl. Acad. Sci. U. S. A. 1974, 71, 2892.
